# Efficacy of CAR‐T‐Cell‐Based Immunotherapies in Patients With Glioma: A Systematic Review and Meta‐Analysis

**DOI:** 10.1111/jcmm.71358

**Published:** 2026-09-25

**Authors:** Seyed Mostafa Mostafavi Zadeh, Sima Kalantari, Shaghayegh Shafiee Dastgerdi, Hossein Sharifi, Mahdi Mohammadi, Raheleh Roudi, Ahmad Shariftabrizi, Abolfazl Kalantari, Davoud Ahmadvand, Maryam Yazdi, Reza Nedaeinia

**Affiliations:** ^1^ Division of Gene Therapy Science Gunma University, Initiative for Advanced Research (GIAR) Maebashi Japan; ^2^ Department of Molecular Imaging, Faculty of Advanced Technologies in Medicine Iran University of Medical Sciences (IUMS) Tehran Iran; ^3^ Iranian Society of Molecular Medicine Tehran Iran; ^4^ Faculty of Physics University of Isfahan Isfahan Iran; ^5^ Department of Radiology, Molecular Imaging Program at Stanford Stanford University Stanford California USA; ^6^ Department of Radiology University of Iowa Hospitals and Clinics Iowa city Iowa USA; ^7^ Child Growth and Development Research Center, Research Institute for Primordial Prevention of Non‐Communicable Disease Isfahan University of Medical Sciences Isfahan Iran; ^8^ Pediatric Inherited Diseases Research Center, Research Institute for Primordial Prevention of Non‐Communicable Disease Isfahan University of Medical Sciences Isfahan Iran

**Keywords:** CAR‐T cells, glioma, immunotherapy, meta‐analysis

## Abstract

Glioma is an aggressive intracranial malignancy associated with a poor prognosis and high mortality. Chimeric antigen receptor (CAR) T‐cell therapy has emerged as a promising immunotherapeutic approach for solid tumours; however, its application in glioma is constrained by several significant challenges. This study conducted a systematic review and meta‐analysis to synthesise current methodologies and critically evaluate the clinical efficacy of CAR‐T‐cell‐based immunotherapies in patients with glioma. A comprehensive literature search was performed across PubMed, Scopus, Web of Science and Embase using MeSH terms along with relevant keywords. The review process adhered to the PRISMA framework, applying clearly defined inclusion and exclusion criteria. Standard and augmented meta‐analyses were employed to evaluate clinical outcomes, associated complications and potential publication bias. The study protocol was prospectively registered in PROSPERO under registration number CRD42022373297. Of 7723 retrieved records, 23 studies encompassing 306 patients with glioma met inclusion criteria. Efficacy analysis of 12 studies (*n* = 153 evaluable patients) demonstrated a pooled overall response rate of 16.9% (95% confidence interval [CI], 8.0%–32.2%), driven by complete responses in 8.1% (95% CI, 3.5%–17.8%) and partial responses in 13.1% (95% CI, 6.3%–25.5%) of patients. Stable disease was achieved in 46.6% (95% CI, 34.3%–59.3%); progressive disease was observed in 43.9% (95% CI, 27.7%–61.5%). Safety evaluation revealed grade ≥ 3 adverse events in 18.6% (95% CI, 10.0%–33.0%) of treated patients (*n* = 69 across 9 studies). Cytokine release syndrome affected 29.7% (95% CI, 9.3%–63.3%) (*n* = 80 across 9 studies), while immune effector cell‐associated neurotoxicity syndrome developed in 12.9% (95% CI, 3.6%–37.0%) (*n* = 59 across 7 studies). This review demonstrated CAR‐T‐cell therapy to have promise in glioma, but evidence remains limited by heterogeneity and small samples. Larger standardised trials, systematic adverse‐event reporting and mechanistic studies on constructs, dosing and combinations are needed. Biomarker discovery will refine patient selection, reduce risk and clarify the therapeutic role of CAR‐T cells in glioma.

## 1 Introduction

Glioma is both the most prevalent and the most aggressive malignant brain tumour in adults, distinguished by rapid proliferation, marked invasiveness, diffuse infiltration of adjacent neural tissue and a dismal prognosis [1, 2, 3, 4]. Globally, the incidence of glioma is estimated at 3.19 cases per 100,000 person‐years, with a clear male predominance (male: female ratio 1.6:1) [5, 6]. The disease most commonly manifests between 55 and 75 years of age, with occurrence in children and young adults remaining uncommon [7, 8, 9].

The diagnosis of glioma is established through neuroimaging, histopathological evaluation and increasingly, molecular profiling techniques [3, 5, 10]. The current standard of care consists of maximal safe surgical resection followed by concurrent radiotherapy and temozolomide chemotherapy, with subsequent adjuvant treatment [11]. However, the tumour's location within the central nervous system, together with the restrictive blood–brain barrier and an immunosuppressive microenvironment, significantly limits therapeutic accessibility and efficacy [6]. Consequently, glioma derives only modest benefit from conventional modalities such as surgery, radiotherapy and chemotherapy. Even with multimodal interventions, the median overall survival remains approximately 12–15 months, and the 5‐year survival rate is below 5% [5, 12].

Chimeric antigen receptor (CAR) T‐cell therapy represents a promising non‐surgical immunotherapy for glioma [13]. Although initially developed for haematological malignancies, it has been applied to glioma due to its capacity to penetrate the blood–brain barrier [14] and counteract glioma‐associated immunosuppression [15]. It is particularly advantageous for unresectable or recurrent glioma [16]. However, the immunosuppressive tumour microenvironment remains a major barrier, limiting T‐cell infiltration, persistence and functional activity, thereby diminishing therapeutic efficacy. Preliminary studies indicate that combining CAR‐T‐cell therapy with gene editing, cytokine supplementation, or immune checkpoint blockade may improve its efficacy [17, 18]. Thus, CAR‐T‐cell therapy represents a compelling strategy to overcome the limitations of current treatment paradigms [6]. Despite its promise, CAR‐T‐cell therapy for glioma must overcome several critical barriers, including tumour heterogeneity, antigen loss, the immunosuppressive microenvironment and the restrictive blood–brain barrier [6]. Current research focuses on optimising CAR‐T‐cell delivery methods, enhancing antigen specificity and developing combination approaches. Moving forward, a critical appraisal of prior immunotherapeutic efforts is essential to understand treatment failure mechanisms and guide the rational design of next‐generation CAR‐T strategies [19].

Several systematic reviews and meta‐analyses have previously examined CAR‐T‐cell therapy in glioma. Jang et al. reported a pooled objective response of 5.1% and a median overall survival of 8.1 months across 63 patients with recurrent glioma, concluding that, while CAR‐T‐cell therapy appears relatively safe, its efficacy remains limited [20]. Agosti et al. reviewed 151 patients across eight studies, highlighting the therapeutic potential of targeting EGFRvIII and IL13Rα2, while emphasising persistent challenges such as antigen escape and tumour heterogeneity [21]. Azzam et al. conducted a meta‐analysis of phase I trials (108 patients), reporting a pooled median overall survival of 6.49 months and noting that complete responses were uncommon [22]. Czyżewski et al. provided a comprehensive synthesis of advances and challenges in this field [16]. Despite these contributions, several questions remain unanswered regarding optimal CAR construct design, delivery routes, patient selection and combination strategies. The present review and meta‐analysis therefore aimed to provide an updated and comprehensive evaluation of CAR‐T‐cell therapy efficacy in glioma, incorporating recent studies published up to 2026, with a particular focus on therapeutic response and survival outcomes.

## 2 Methods

### 2.1 Search Strategy

This systematic review and meta‐analysis followed the Preferred Reporting Items for Systematic Reviews and Meta‐Analyses (PRISMA) 2020 guidelines [23]. The protocol was prospectively registered with PROSPERO (https://www.crd.york.ac.uk/PROSPERO/view/CRD42022373297). A systematic search of the PubMed, Embase, Scopus, Web of Science and ProQuest databases was performed. The date range was set from January 1, 1992, to June 3, 2026, to capture the full evolution of CAR‐T‐cell research in gliomas; we acknowledge that this represents an arbitrary date restriction without formal methodological justification. We constructed the search strategy according to the PICO framework (patients: patients with glioma; intervention: CAR‐T‐cell‐based immunotherapies; comparison: standard of care or other treatments; outcomes: efficacy and safety measures). Keywords related to the patient (glioma) and intervention (adoptive cellular immunotherapy, CAR‐T‐cell therapy) components were used, along with appropriate Boolean operators. Search strategies utilised free‐text terms and database‐specific syntax. The specific search strategies for each database are summarised in Table S1. No language restrictions were applied. Additionally, we manually screened the reference lists of included studies and relevant reviews to identify additional eligible reports.

### 2.2 Study Selection

We included original studies that evaluated the efficacy and safety of CAR‐T‐cell‐based immunotherapies in patients with glioma, irrespective of treatment line or prior therapies. This study followed a rigorous selection process that adhered to established standards. High‐quality evidence was ensured by including only studies published in peer‐reviewed journals. Irrelevant articles, reviews, case reports/series, conference abstracts, animal studies, letters/editorials and book chapters were identified and excluded during the screening process.

**FIGURE 1:**
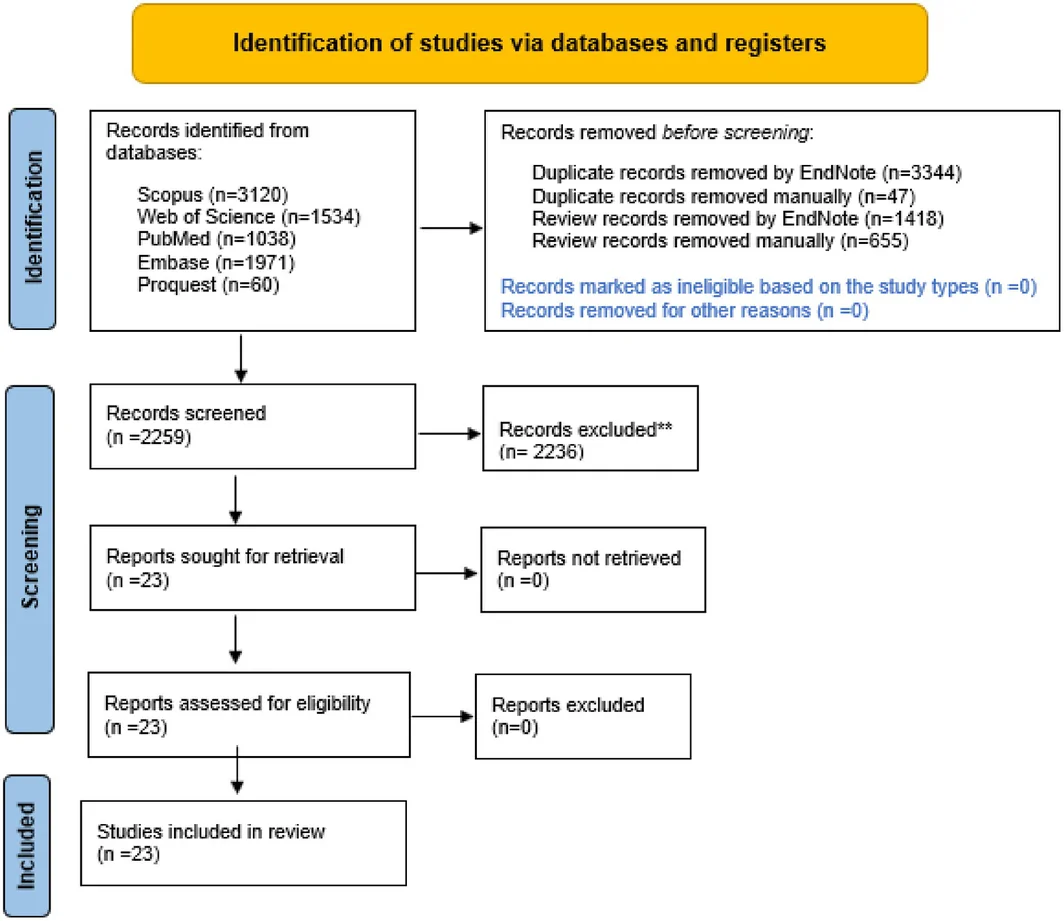
PRISMA flow diagram of study identification, screening, eligibility assessment and inclusion.

The initially retrieved articles were imported into EndNote software. After deduplication using the Bramer method [24], which systematically identifies and removes duplicate records while preserving unique entries, the remaining unique studies were independently reviewed by two authors (S.S.D. and H.S.) based on their titles and abstracts. The articles selected in this step were then assessed based on their full texts, and the works selected in this second step were reviewed by the same two authors. To ensure a comprehensive search, we used major databases and manually searched the reference lists of relevant articles. Any discrepancies during the screening process were resolved by a third reviewer (S.K.), and the final selection of studies was reached through consensus. The study selection process is illustrated in Figure 1.

### 2.3 Data Extraction and Risk of Bias Assessment

Two reviewers independently extracted data from the eligible full‐text reports using a standardised data‐extraction form. Extracted variables included author, publication year, country, study design and trial phase, patient population and tumour characteristics, target antigen, CAR‐T construct, route and dose of administration, pre‐treatment history, number of enrolled, treated and outcome‐evaluable participants, duration of follow‐up, response criteria, complete response (CR), partial response (PR), stable disease (SD), progressive disease (PD), grade ≥ 3 adverse events, cytokine release syndrome (CRS), immune effector cell‐associated neurotoxicity syndrome (ICANS) and survival outcomes where available. Disagreements were resolved through discussion and, where required, adjudication by a third reviewer.

When multiple reports appeared to describe overlapping trial cohorts, cohort identity, CAR‐T construct, data‐cutoff date and outcome‐evaluable sample size were reviewed to avoid double counting of participants within each outcome‐specific meta‐analysis. Where duplication was identified, the most complete or updated report was prioritised for quantitative synthesis, while related reports were retained for qualitative synthesis when they provided complementary clinical or translational information.

Methodological quality and risk of bias were assessed using the Joanna Briggs Institute (JBI) Critical Appraisal Checklist for Case Series. The checklist evaluates 10 domains addressing clarity of inclusion criteria, reliability and validity of condition ascertainment, consecutive and complete inclusion of participants, reporting of demographic and clinical characteristics, outcome and follow‐up reporting, study setting and appropriateness of statistical analysis. Each item was rated as “Yes,” “No,” “Unclear,” or “Not applicable.” For descriptive purposes, “Yes” responses were summarised across the 10 domains; however, no validated numerical cutoff was applied, and no study was excluded based on the JBI appraisal. Detailed item‐level assessments are presented in Table S2.

### 2.4 Meta‐Analysis

Outcome‐specific meta‐analyses were conducted for overall response (OR; defined as CR plus PR), CR, PR, SD, PD, grade ≥ 3 adverse events, CRS and ICANS. For each study, the event proportion was calculated as the number of participants experiencing the outcome divided by the number of outcome‐evaluable participants.

Because several outcomes involved sparse data and studies with zero or complete events, proportions were analysed on the logit scale. A continuity correction of 0.5 was applied to both the event and non‐event cells only when a study reported zero events or events in all participants. Random‐effects models were fitted using restricted maximum likelihood (REML), and pooled estimates with 95% confidence intervals (CIs) were back‐transformed to the proportion scale for reporting.

Between‐study heterogeneity was assessed using Cochran's Q statistic, *I* ^2^ and τ^2^. Forest plots were generated for OR, PR, SD, PD, grade ≥ 3 adverse events, CRS and ICANS. All primary meta‐analyses were conducted using Stata/SE version 17.0 (StataCorp LLC, College Station, TX, USA).

### 2.5 Subgroup and Sensitivity Analysis

Exploratory subgroup analyses were conducted according to CAR‐T target antigen or marker type, including HER2, IL‐13Rα2, EGFRvIII, GD2, EphA2, B7‐H3, EGFR806, CLTX‐CAR and dual‐target CAR‐T constructs. Pooled estimates, 95% CIs and heterogeneity statistics were calculated within each marker subgroup when at least two reports were available. Subgroups represented by a single report were summarised descriptively without an *I* ^2^ estimate. Given the small number of studies within most subgroups, these analyses were considered exploratory, and no formal between‐subgroup hypothesis testing was performed.

Leave‐one‐out sensitivity analyses were performed for each outcome by sequentially omitting one report and recalculating the pooled proportion. To provide a computationally stable sensitivity analysis across sparse outcomes, the leave‐one‐out analyses used random‐effects DerSimonian‐Laird models on the logit‐proportion scale, with back‐transformation to proportions for interpretation. Changes in pooled estimates were expressed in percentage points relative to the full‐data primary estimate. Leave‐one‐out results are summarised in Table S3 and Figures [Link], [Link], [Link], [Link], [Link], [Link], [Link], [Link].

### 2.6 Publication Bias Assessment

Potential small‐study effects, which may reflect publication bias but are not diagnostic of it, were assessed using contour‐enhanced funnel plots and Egger's regression test. Formal testing was undertaken only for outcomes reported by at least 10 studies. Egger's regression was performed on logit‐transformed proportions using a random‐effects meta‐regression approach. A continuity correction of 0.5 was applied only to studies reporting zero or complete events. Outcomes reported by fewer than 10 studies, including grade ≥ 3 adverse events, CRS and ICANS, were not formally assessed because funnel‐plot asymmetry tests are underpowered in small meta‐analyses. All statistical tests were two‐sided, with *p* < 0.05 considered statistically significant.

## 3 Results

### 3.1 Study Characteristics

A total of 23 studies met the eligibility criteria and were included in this systematic review and meta‐analysis [25, 26, 27, 28, 29, 30, 31, 32, 33, 34, 35, 36, 37, 38, 39, 40, 41, 42, 43, 44, 45, 46]. The characteristics of the included studies are summarised in Table 1. The publication years ranged from 2015 to 2026. The included studies were conducted across three countries: the United States (*n* = 18), China (*n* = 4) and South Korea (*n* = 1). Regarding study design, 21 studies were phase I clinical trials, 1 was a phase I/IIa trial [28], and 1 was a translational/biomarker substudy [45].

**TABLE 1:** Summary of characteristics of included studies.

First author (Year)	Country	Study ID	Phase	Sample size (*n*)	Male (%)	Glioma subtype	Target antigen	Route of administration	Concomitant therapy
Ahmed, N. (2017)	USA	NCT01109095	Phase 1	17 enrolled; 16 evaluable	53	Progressive HER2‐positive glioblastoma	HER2	IV	Surgery, RT, TMZ
Brown, C. (2024)	USA	NCT02208362	Phase 1	92 enrolled; 65 treated; 58 evaluable	62	Recurrent HGG (grade 3–4)	IL‐13Rα2	Locoregional (ICT, ICV, dual)	Surgery, bevacizumab
Brown, C. (2022)	USA	NCT01082926	Phase 1	6	67	rGBM (unresectable)	IL‐13Rα2	IT (intracranial)	Dexamethasone, IL‐2
Goff, S. (2019)	USA	NCT01454596	Phase 1	18	83	rGBM (EGFRvIII+)	EGFRvIII	IV	Lymphodepletion (Cy/Flu), IL‐2
Lim, J. (2021)	South Korea	KCT0003815	Phase I/IIa	14	43	rGBM	NA (NK/T cells)	IV	Standard therapy
Lin, F. Y. (2024)	USA	NCT04099797	Phase 1	11 treated	55	Paediatric DMG/ATRT	GD2	IV	RT, chemo, lymphodepletion
Lin, Q. (2021)	China	NCT03423992	Phase 1	3	67	rGBM	EphA2	IV	Lymphodepletion (Cy/Flu)
Monje, M. (2025)	USA	NCT04196413	Phase 1	13 enrolled; 11 treated	36	H3K27M‐mutant DMG	GD2	Sequential IV + ICV	Lymphodepletion (Cy/Flu), RT
O'Rourke, D. M. (2017)	USA	NCT02209376	Phase 1	10	50	rGBM (EGFRvIII+)	EGFRvIII	IV	Surgery, RT, TMZ
Vitanza, N. A. (2023)	USA	NCT04185038	Phase 1	5 enrolled; 3 evaluable	40	DIPG	B7‐H3	IV	RT
Bagley, S. (2024)	USA	NCT01109095	Phase 1	7	71	Newly diagnosed GBM (EGFRvIII+)	EGFRvIII	IV	RT, pembrolizumab
Choi, B. D. (2024)	USA	NCT05660369	Phase 1	3	67	rGBM (EGFRvIII+)	EGFRvIII + EGFR (TEAM)	ICV	Surgery, RT, TMZ
Brown, C. E. (2015)	USA	NCT00730613	Phase 1	3	33	rGBM	IL‐13Rα2	Intracavitary/IT	Surgery, BCNU, RT, TMZ
Gu, J. (2025)	China	NA	NA	6	83	rGBM/DMG (grade IV)	PSMA + GD2	IV	Lymphodepletion (Cy/Flu), RT, TMZ
Gust, J. (2025)	USA	NCT03638167	Phase 1	11 enrolled; 4 treated	50	Paediatric HGG/ATRT	EGFR (mAb806)	Locoregional (IT/ICV)	None
Liu, Z. (2023)	China	NCT03170141	Phase 1	8	50	rGBM	GD2	IV ± IC	Lymphodepletion (Cy/Flu)
Barish, M. E. (2025)	USA	NCT04214392	Phase 1	5 enrolled; 4 treated	25	rGBM	MMP‐2 (CLTX‐CAR)	ICT (intracavitary/IT)	None
Vitanza, N. A. (2025)	USA	NCT04185038	Phase 1	23 enrolled; 21 treated	39	DIPG/DMG	B7‐H3	ICV	None, RT
Majzner, R. G. (2022)	USA	NCT04196413	Phase 1	4	25	H3K27M‐mutant DMG	GD2	IV ± ICV	Lymphodepletion (Cy/Flu) ± ICV
Bagley, S. J. (2024)	USA	NCT05168423	Phase 1	6	100	rGBM (EGFR amp)	EGFR (806) + IL‐13Rα2	IT (ICV)	None
Bagley, S. J. (2025)	USA	NCT05168423	Phase 1	18	83	rGBM (EGFR amp)	EGFR (806) + IL‐13Rα2	ICV	None
Tang, O. Y. (2022)	USA	NCT02209376	Translational	10 enrolled; 9 analysed	56	rGBM (EGFRvIII+)	EGFRvIII	IV	Standard therapy
Li, X. (2026)	China	ChiCTR2000028801	Phase 1	5	20	rHGG (GBM)	IL‐13Rα2	IT (lumbar puncture)	None

A total of 306 patients with glioma were enrolled across the included studies, with sample sizes ranging from 3 to 92 patients per study. Among the treated patients, the median age across studies ranged from 6 to 72 years, with 12 studies exclusively enrolling adult patients (≥ 18 years) and 4 studies exclusively enrolling paediatric patients (< 18 years). The remaining 7 studies included mixed adult and paediatric populations. The proportion of male patients ranged from 25% to 100%, with a weighted average of approximately 55%, while female patients constituted the remainder. Regarding glioma subtypes, 20 studies included patients with glioblastoma (WHO grade IV), 3 studies included patients with anaplastic astrocytoma (WHO grade III) or other high‐grade gliomas, 5 studies included paediatric patients with diffuse midline glioma (DMG) or diffuse intrinsic pontine glioma (DIPG), and 22 studies included patients with recurrent or progressive disease. One study [34] included patients with newly diagnosed EGFRvIII‐positive glioblastoma.

The CAR‐T‐cell constructs varied considerably across studies. The most commonly targeted antigens were EGFRvIII (*n* = 5 studies: [27, 32, 34, 35, 45] [combined with EGFR]), IL‐13Rα2 (*n* = 5 studies: [26, 36, 43, 44] [combined with EGFR], [46]), GD2 (*n* = 4 studies: [29, 31, 39, 42]), B7‐H3 (*n* = 2 studies: [33, 41]), HER2 (*n* = 1 study: [25]), EphA2 (*n* = 1 study: [30]) and MMP‐2 via CLTX‐CAR (*n* = 1 study: [40]). Additionally, 3 studies utilised dual‐targeted or bivalent CARs, including EGFR/IL‐13Rα2 [43, 44], PSMA/GD2 [37] and EGFRvIII/EGFR via TEAM technology [35]. One study [28] utilised expanded natural killer (NK) and T lymphocytes rather than CAR‐T cells. Regarding CAR generations, 8 studies utilised second‐generation CARs (containing 4‐1BB or CD28 costimulatory domains), 3 utilised third‐generation CARs [27, 30, 36] [or first‐generation zetakine], 2 utilised fourth‐generation safety‐designed CARs (4SCAR‐T) with inducible suicide genes [37, 39], 2 utilised first‐generation CARs (IL13‐zetakine) [26, 36], 1 utilised an allogeneic universal CAR‐T with TCR and HLA‐I double knockout [46], and 1 utilised a CAR with a secreted T‐cell‐engaging antibody molecule (TEAM) [35]. The remaining studies either did not specify CAR generation or utilised non‐CAR expanded immune cells. The routes of administration included intravenous (*n* = 11), intratumoral/intracavitary (*n* = 4), intraventricular/intracerebroventricular (*n* = 7), intrathecal (*n* = 2) and locoregional combinations (*n* = 3). Regarding concomitant therapies, 7 studies combined CAR‐T‐cell therapy with lymphodepleting chemotherapy (fludarabine/cyclophosphamide), 1 combined with pembrolizumab (anti‐PD‐1) [34], 2 combined with IL‐2 [26, 27] and the remainder used CAR‐T cells as monotherapy. Three studies [26, 38, 40] did not use lymphodepleting chemotherapy.

The median follow‐up duration, where reported, ranged from 1.5 to 18.7 months [44]. Outcome measures reported included overall survival in 12 studies, progression‐free survival (PFS) in 6 studies, objective response rate (ORR) in 14 studies, disease control rate (DCR) in 7 studies, and treatment‐related adverse events (AEs) in all included studies.

### 3.2 Quality Assessment

All 23 included reports were appraised using the JBI Critical Appraisal Checklist for Case Series. The mean number of affirmative responses was 8.9 out of 10 (standard deviation 0.5), with a median of 9 and a range of 7–9. Most studies clearly reported eligibility criteria, diagnostic confirmation, demographic and clinical characteristics, treatment outcomes, follow‐up information, and the study setting.

Explicit consecutive recruitment was not reported in any of the included reports. This should be interpreted primarily as incomplete reporting of recruitment procedures rather than definitive evidence of selective participant inclusion. Complete inclusion of the analysed cohort was confirmed in 21 reports; one preliminary report included only a limited evaluable subgroup, and one translational analysis excluded a participant from a correlational analysis due to an outlying progression‐free survival value. Appropriate statistical analysis was judged to be adequately reported in 22 studies. No study was excluded based on methodological‐quality appraisal. Detailed JBI item‐level assessments are provided in Table S2.

### 3.3 Meta‐Analysis

**FIGURE 2:**
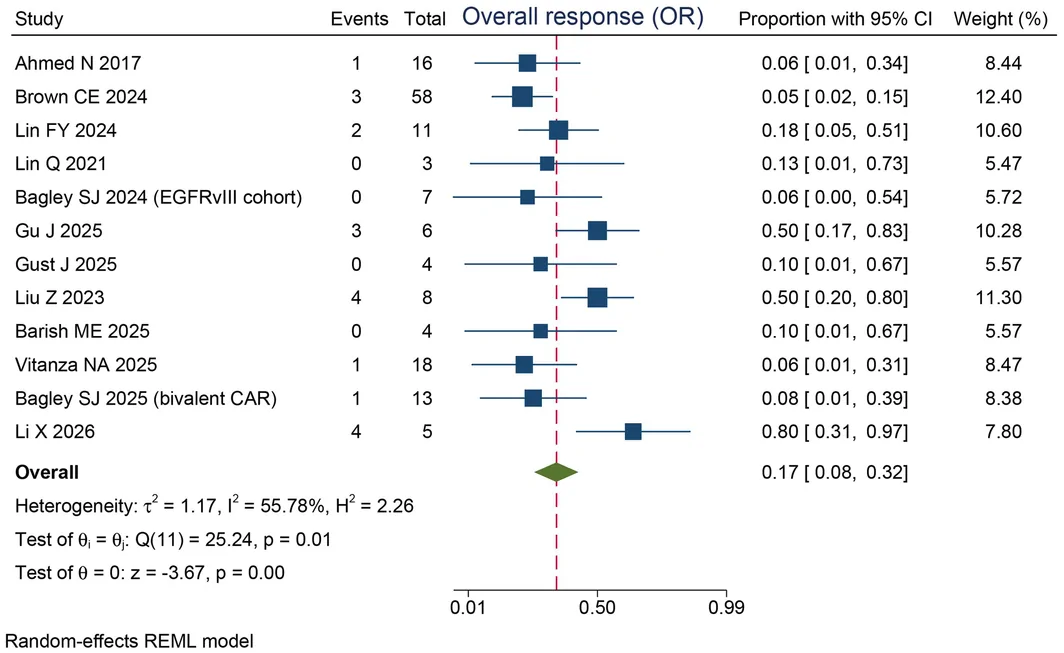
Forest plot of the pooled proportion of overall response (OR), defined as complete response plus partial response, following CAR‐T‐cell‐based immunotherapy in patients with glioma. Random‐effects REML meta‐analysis of logit‐transformed proportions; pooled estimates were back‐transformed to the proportion scale. Squares represent study‐specific proportions, horizontal lines indicate 95% CIs, and the diamond represents the pooled estimate.

**FIGURE 3:**
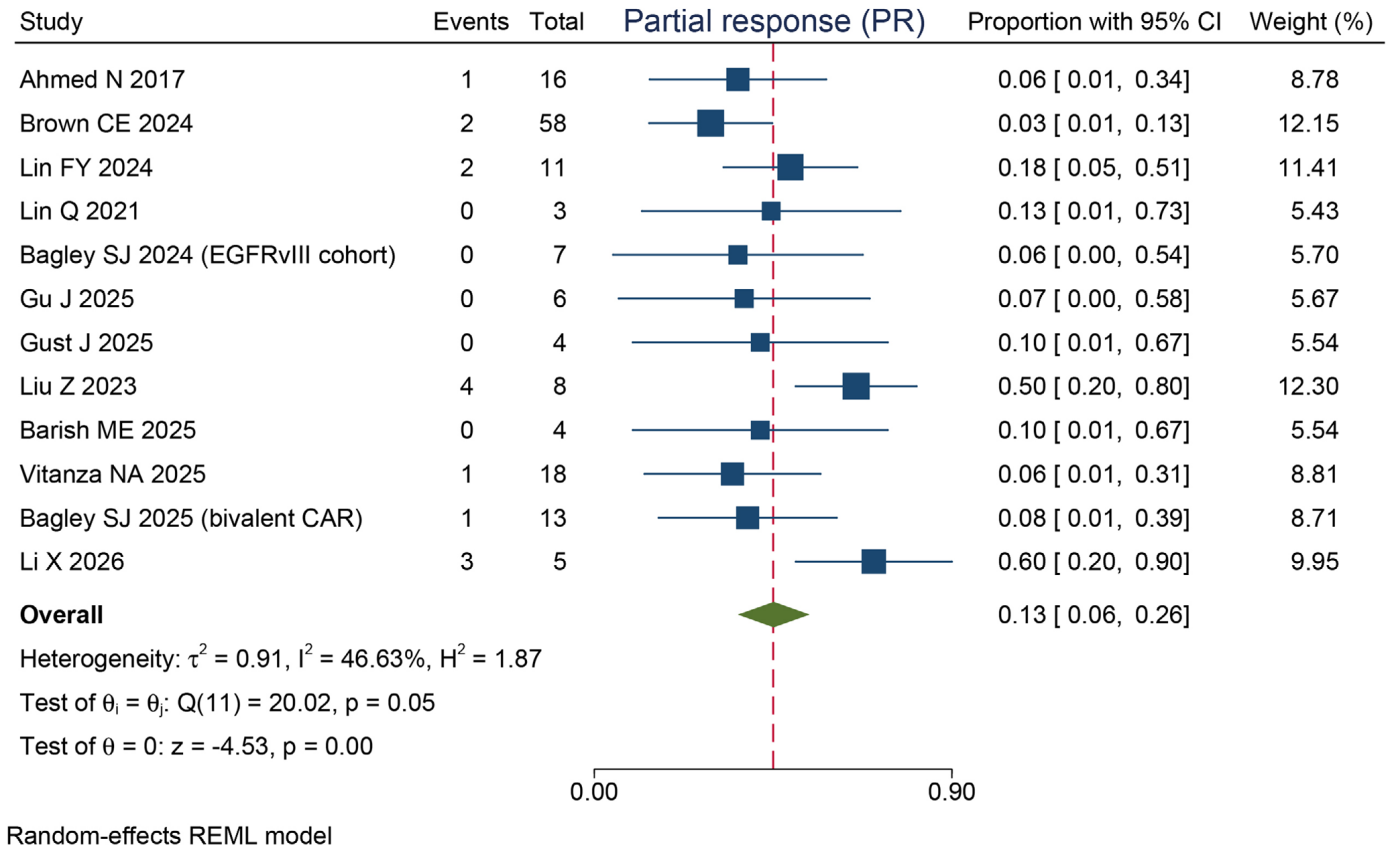
Forest plot of the pooled proportion of partial response (PR) following CAR‐T‐cell‐based immunotherapy in patients with glioma. Random‐effects REML meta‐analysis of logit‐transformed proportions. Squares represent study‐specific proportions, horizontal lines indicate 95% CIs, and the diamond represents the pooled estimate.

Twelve reports comprising 153 outcome‐evaluable participants contributed to the overall response analysis. The pooled OR proportion was 16.9% (95% CI, 8.0%–32.2%), with moderate heterogeneity (*I* ^2^ = 55.8%; τ^2^ = 1.17; Figure 2). The pooled CR proportion, reported in Table 2, was 8.1% (95% CI, 3.5%–17.8%; I^2^ = 32.5%). The pooled PR proportion was 13.1% (95% CI, 6.3%–25.5%), with moderate heterogeneity (*I* ^2^ = 46.6%; Figure 3).

**TABLE 2:** Exploratory subgroup analyses of efficacy and safety outcomes according to CAR‐T target antigen or marker type.

Outcome	Marker type	No. of studies	No. of patients	Proportion [95% CI]	*I* ^2^ (%)
OR	B7‐H3 (CD276)	1	18	0.056 [0.010, 0.258]	—
	CLTX‐CAR (MMP‐2 surrogate)	1	4	0.000 [0.000, 0.490]	—
	EGFR806	1	4	0.000 [0.000, 0.490]	—
	EGFR806 + IL‐13Rα2	1	13	0.077 [0.014, 0.333]	—
	EGFRvIII	1	7	0.000 [0.000, 0.354]	—
	EphA2	1	3	0.000 [0.000, 0.561]	—
	GD2	2	19	0.328 [0.101, 0.681]	50.9
	HER2	1	16	0.063 [0.011, 0.283]	—
	IL‐13Rα2	2	63	0.296 [0.006, 0.966]	91.3
	Overall	12	153	0.169 [0.080, 0.322]	55.8
	PSMA + GD2	1	6	0.500 [0.188, 0.812]	—
CR	B7‐H3 (CD276)	1	18	0.000 [0.000, 0.176]	—
	CLTX‐CAR (MMP‐2 surrogate)	1	4	0.000 [0.000, 0.490]	—
	EGFR806	1	4	0.000 [0.000, 0.490]	—
	EGFR806 + IL‐13Rα2	1	13	0.000 [0.000, 0.228]	—
	EGFRvIII	1	7	0.000 [0.000, 0.354]	—
	EphA2	1	3	0.000 [0.000, 0.561]	—
	GD2	2	19	0.048 [0.007, 0.274]	0.0
	HER2	1	16	0.000 [0.000, 0.194]	—
	IL‐13Rα2	2	63	0.060 [0.005, 0.461]	67.9
	Overall	12	153	0.081 [0.035, 0.178]	32.5
	PSMA + GD2	1	6	0.500 [0.188, 0.812]	—
PR	B7‐H3 (CD276)	1	18	0.056 [0.010, 0.258]	—
	CLTX‐CAR (MMP‐2 surrogate)	1	4	0.000 [0.000, 0.490]	—
	EGFR806	1	4	0.000 [0.000, 0.490]	—
	EGFR806 + IL‐13Rα2	1	13	0.077 [0.014, 0.333]	—
	EGFRvIII	1	7	0.000 [0.000, 0.354]	—
	EphA2	1	3	0.000 [0.000, 0.561]	—
	GD2	2	19	0.328 [0.101, 0.681]	50.9
	HER2	1	16	0.063 [0.011, 0.283]	—
	IL‐13Rα2	2	63	0.182 [0.006, 0.896]	90.3
	Overall	12	153	0.131 [0.063, 0.255]	46.6
	PSMA + GD2	1	6	0.000 [0.000, 0.390]	—
SD	B7‐H3 (CD276)	1	18	0.833 [0.608, 0.942]	—
	CLTX‐CAR (MMP‐2 surrogate)	1	4	0.750 [0.301, 0.954]	—
	EGFR806	1	4	0.250 [0.046, 0.699]	—
	EGFR806 + IL‐13Rα2	1	13	0.615 [0.355, 0.823]	—
	EGFRvIII	1	7	0.000 [0.000, 0.354]	—
	EphA2	1	3	0.333 [0.061, 0.792]	—
	GD2	2	19	0.301 [0.075, 0.696]	51.5
	HER2	1	16	0.438 [0.231, 0.668]	—
	IL‐13Rα2	2	63	0.426 [0.288, 0.578]	5.0
	Overall	12	153	0.466 [0.343, 0.593]	36.3
	PSMA + GD2	1	6	0.500 [0.188, 0.812]	—
PD	B7‐H3 (CD276)	1	18	0.111 [0.031, 0.328]	—
	CLTX‐CAR (MMP‐2 surrogate)	1	4	0.250 [0.046, 0.699]	—
	EGFR806	1	4	0.750 [0.301, 0.954]	—
	EGFR806 + IL‐13Rα2	1	13	0.308 [0.127, 0.576]	—
	EGFRvIII	2	24	0.940 [0.750, 0.988]	0.0
	EphA2	1	3	0.667 [0.208, 0.939]	—
	GD2	2	19	0.368 [0.187, 0.597]	0.0
	HER2	1	16	0.500 [0.280, 0.720]	—
	IL‐13Rα2	2	63	0.319 [0.050, 0.806]	60.9
	Overall	13	170	0.439 [0.277, 0.615]	64.8
	PSMA + GD2	1	6	0.000 [0.000, 0.390]	—
CRS	B7‐H3 (CD276)	1	21	0.000 [0.000, 0.155]	—
	CLTX‐CAR (MMP‐2 surrogate)	1	4	0.000 [0.000, 0.490]	—
	EGFR806	1	4	0.000 [0.000, 0.490]	—
	EGFR806 + IL‐13Rα2	1	18	1.000 [0.824, 1.000]	—
	EGFRvIII	1	7	0.000 [0.000, 0.354]	—
	EphA2	1	3	0.667 [0.208, 0.939]	—
	GD2	1	11	0.545 [0.280, 0.787]	—
	IL‐13Rα2	1	6	0.000 [0.000, 0.390]	—
	Overall	9	80	0.297 [0.093, 0.633]	70.4
	PSMA + GD2	1	6	0.500 [0.188, 0.812]	—
ICANS	B7‐H3 (CD276)	1	21	0.000 [0.000, 0.155]	—
	EGFRvIII	1	7	0.000 [0.000, 0.354]	—
	EphA2	1	3	0.000 [0.000, 0.561]	—
	GD2	1	11	0.636 [0.354, 0.848]	—
	IL‐13Rα2	2	11	0.077 [0.011, 0.391]	0.0
	Overall	7	59	0.129 [0.036, 0.370]	52.8
	PSMA + GD2	1	6	0.000 [0.000, 0.390]	—

Abbreviations: CI, confidence interval; CR, complete response; CRS, cytokine release syndrome; ICANS, immune effector cell associated neurotoxicity syndrome; OR, overall response; PD, progressive disease; PR, partial response; SD, stable disease.

**FIGURE 4:**
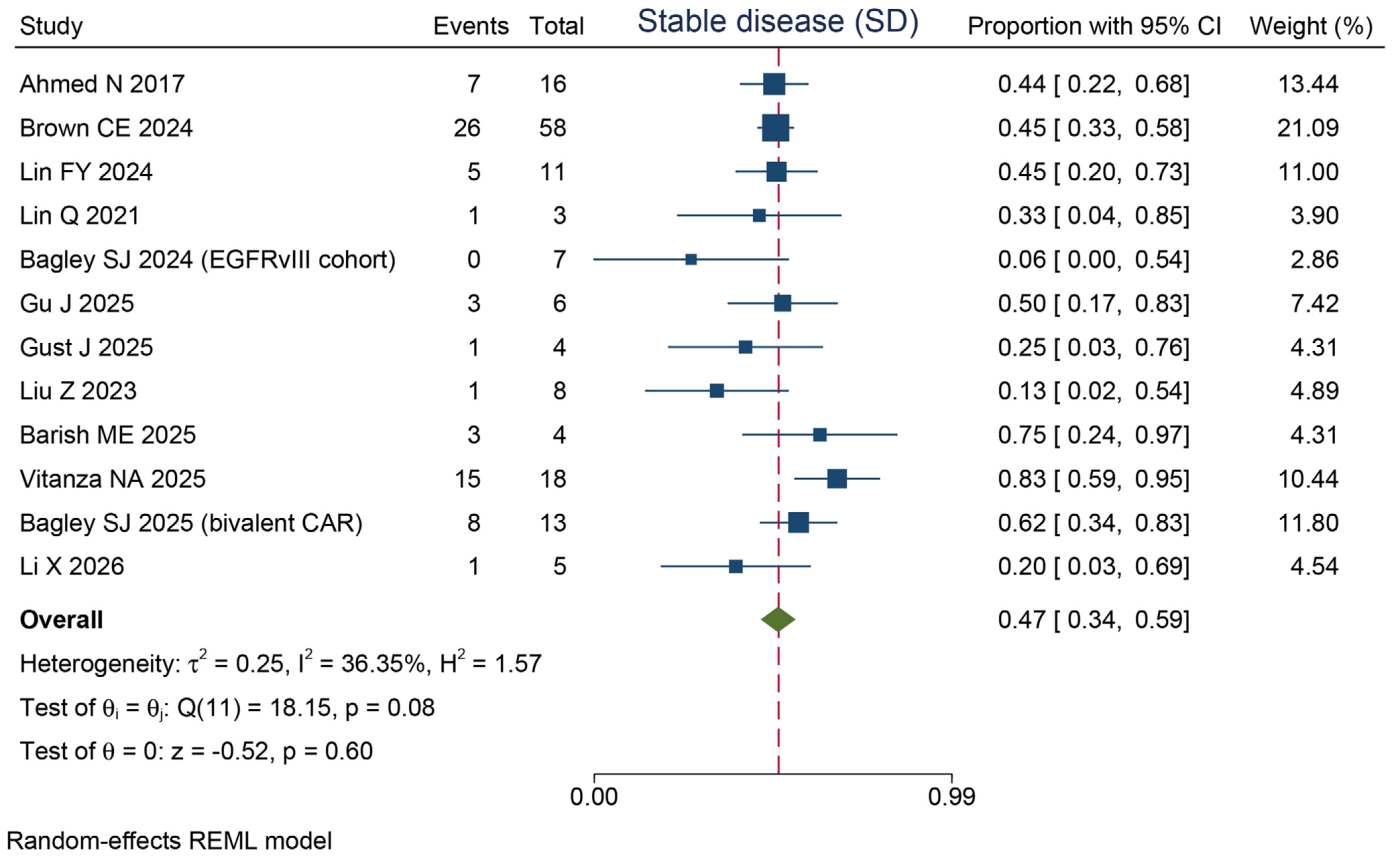
Forest plot of the pooled proportion of stable disease (SD) following CAR‐T‐cell‐based immunotherapy in patients with glioma. Random‐effects REML meta‐analysis of logit‐transformed proportions. Squares represent study‐specific proportions, horizontal lines indicate 95% CIs, and the diamond represents the pooled estimate.

**FIGURE 5:**
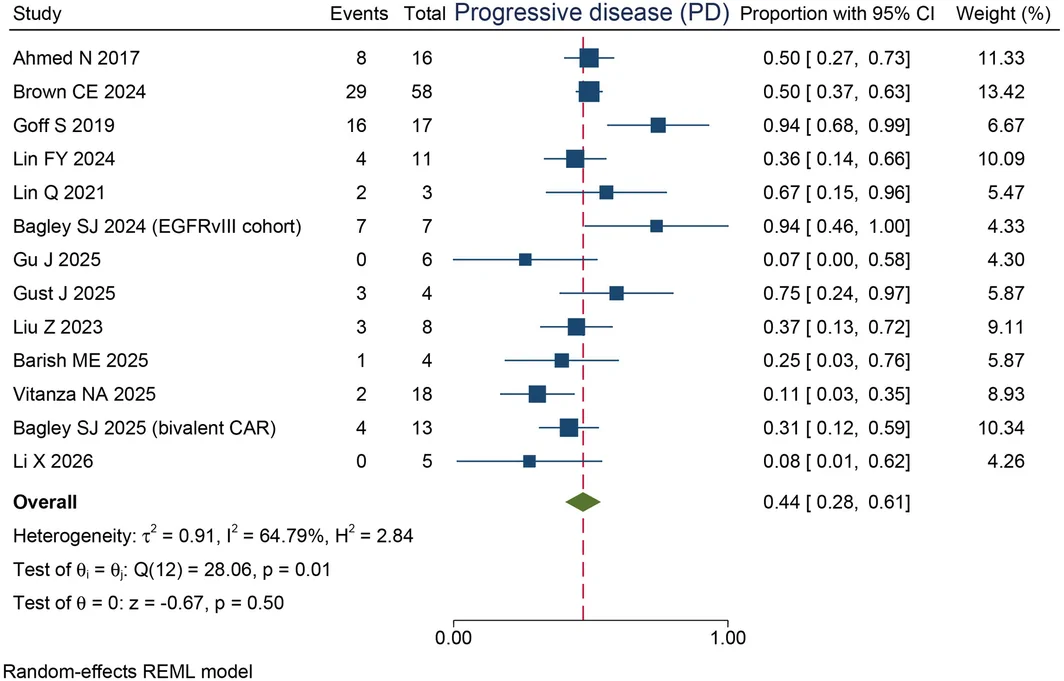
Forest plot of the pooled proportion of progressive disease (PD) following CAR‐T‐cell‐based immunotherapy in patients with glioma. Random‐effects REML meta‐analysis of logit‐transformed proportions. Squares represent study‐specific proportions, horizontal lines indicate 95% CIs, and the diamond represents the pooled estimate.

The pooled SD proportion across 12 reports and 153 participants was 46.6% (95% CI, 34.3%–59.3%), with low‐to‐moderate heterogeneity (*I* ^2^ = 36.4%; Figure 4). Progressive disease was reported in 13 studies including 170 participants, yielding a pooled proportion of 43.9% (95% CI, 27.7%–61.5%), with substantial heterogeneity (*I* ^2^ = 64.8%; Figure 5).

**FIGURE 6:**
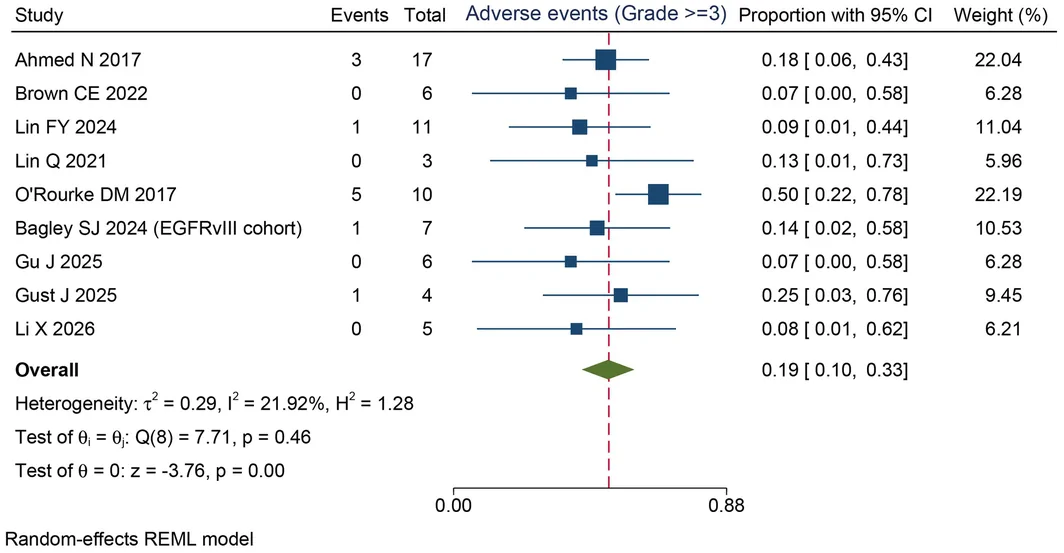
Forest plot of the pooled proportion of grade ≥ 3 adverse events following CAR‐T‐cell‐based immunotherapy in patients with glioma. Random‐effects REML meta‐analysis of logit‐transformed proportions. Squares represent study‐specific proportions, horizontal lines indicate 95% CIs, and the diamond represents the pooled estimate.

**FIGURE 7:**
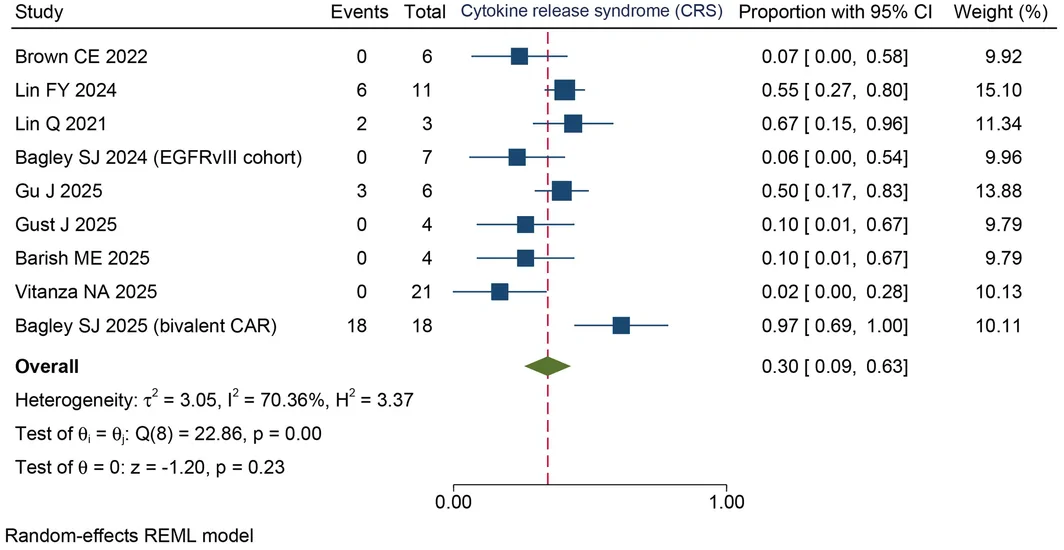
Forest plot of the pooled proportion of cytokine release syndrome (CRS) following CAR‐T‐cell‐based immunotherapy in patients with glioma. Random‐effects REML meta‐analysis of logit‐transformed proportions. Squares represent study‐specific proportions, horizontal lines indicate 95% CIs, and the diamond represents the pooled estimate.

**FIGURE 8:**
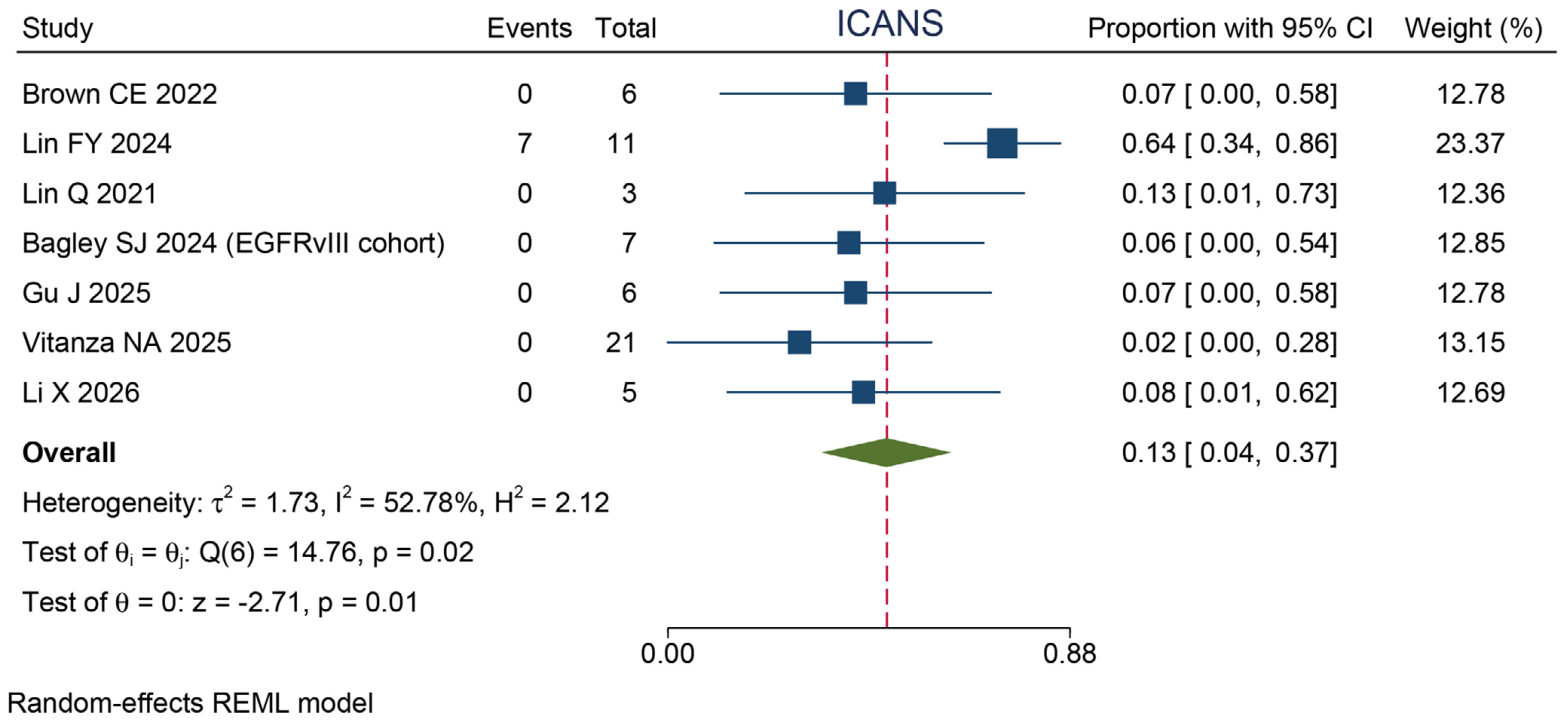
Forest plot of the pooled proportion of immune effector cell‐associated neurotoxicity syndrome (ICANS) following CAR‐T‐cell‐based immunotherapy in patients with glioma. Random‐effects REML meta‐analysis of logit‐transformed proportions. Squares represent study‐specific proportions, horizontal lines indicate 95% CIs, and the diamond represents the pooled estimate.

Nine reports including 69 participants contributed to the analysis of grade ≥ 3 adverse events. The pooled proportion was 18.6% (95% CI, 10.0%–33.0%), with low heterogeneity (*I* ^2^ = 21.9%; Figure 6). CRS was reported in nine studies including 80 participants, with a pooled proportion of 29.7% (95% CI, 9.3%–63.3%) and substantial heterogeneity (*I* ^2^ = 70.4%; Figure 7). Seven reports including 59 participants reported ICANS, yielding a pooled proportion of 12.9% (95% CI, 3.6%–37.0%), with moderate heterogeneity (*I* ^2^ = 52.8%; Figure 8).

### 3.4 Subgroup Analysis

Exploratory subgroup analyses by target antigen are summarised in Table 2. Overall response estimates varied across CAR‐T target groups, although most subgroups included only one report and should not be interpreted as formal comparative evidence. Among marker categories represented by at least two reports, pooled OR proportions were 32.8% for GD2‐targeted CAR‐T‐cell therapy (95% CI, 10.1%–68.1%; *I* ^2^ = 50.9%) and 29.6% for IL‐13Rα2‐targeted CAR‐T‐cell therapy (95% CI, 0.6%–96.6%; *I* ^2^ = 91.3%).

The pooled SD proportion was 42.6% for IL‐13Rα2‐targeted CAR‐T‐cell therapy, with low heterogeneity across the two available reports (*I* ^2^ = 5.0%). In contrast, PD was particularly frequent in the EGFRvIII subgroup, with a pooled proportion of 94.0% across two reports (95% CI, 75.0%–98.8%; *I* ^2^ = 0%). CRS and ICANS estimates were based predominantly on single‐study marker subgroups; therefore, these results should be regarded as descriptive and hypothesis‐generating rather than comparative.

### 3.5 Sensitivity Analysis

Leave‐one‐out sensitivity analyses indicated that no individual report materially altered the overall direction or interpretation of the pooled results. The pooled OR proportion ranged from 13.9% to 19.8% after sequential omission of individual reports; corresponding ranges were 5.5%–10.1% for CR, 10.4%–15.9% for PR, 43.7%–49.1% for SD and 39.7%–47.9% for PD. The pooled proportions for grade ≥ 3 adverse events, CRS and ICANS ranged from 13.6% to 20.3%, 22.6%–37.4% and 6.5%–16.7%, respectively.

The greatest absolute changes were observed after omission of [41] for CRS (+7.7 percentage points) and [29] for ICANS (−6.4 percentage points). Exclusion of [41] reduced heterogeneity for SD to near‐zero levels (*I* ^2^ = 1.4%), while exclusion of [29] reduced ICANS heterogeneity to 0%. These findings indicate that selected studies contributed to between‐study variability but did not exert a dominant influence on the pooled conclusions. Full numerical results are provided in Table S3, and graphical leave‐one‐out analyses are shown in Figures [Link], [Link], [Link], [Link], [Link], [Link], [Link], [Link].

### 3.6 Publication Bias

Egger's regression tests were performed for OR, CR, PR, SD and PD because each outcome was reported by at least 10 studies. No statistically significant evidence of small‐study effects was identified for OR (β_1_ = −0.793, *p* = 0.590), CR (β_1_ = −2.720, *p* = 0.093), PR (β_1_ = −1.250, *p* = 0.368), SD (β_1_ = −1.309, *p* = 0.131), or PD (β_1_ = 0.250, *p* = 0.809).

**FIGURE 9:**
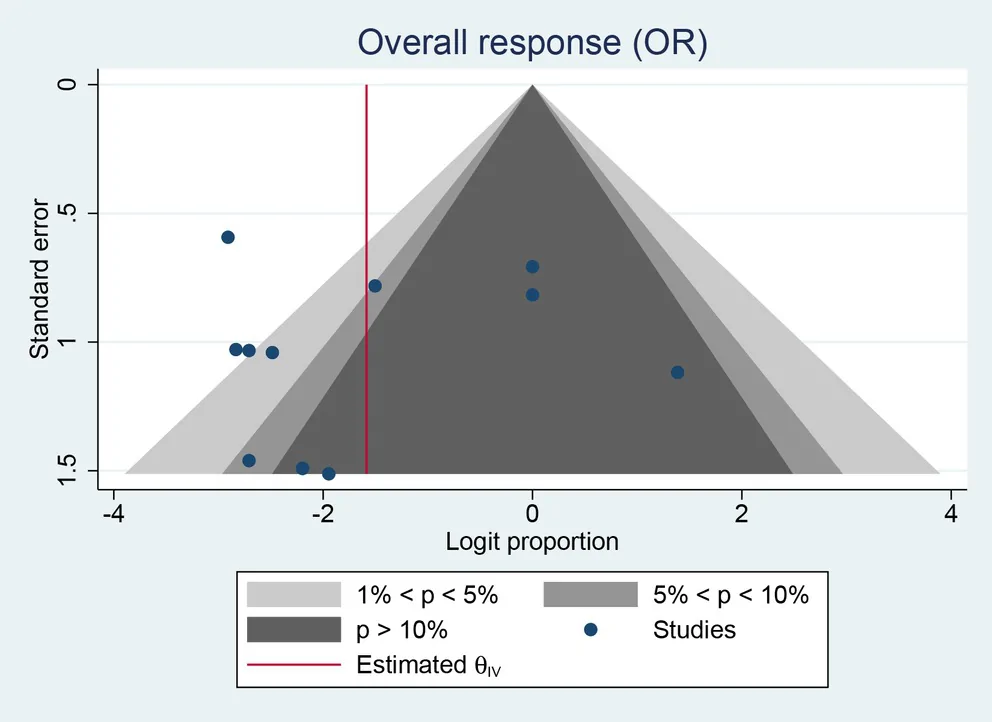
Contour‐enhanced funnel plot for overall response following CAR‐T‐cell‐based immunotherapy in patients with glioma. The plot displays logit‐transformed study‐specific proportions against their standard errors. The vertical line denotes the inverse‐variance random‐effects pooled estimate and contour regions represent nominal two‐sided significance thresholds.

Visual inspection of the contour‐enhanced funnel plot for OR did not reveal marked asymmetry (Figure 9). Formal testing was not performed for grade ≥ 3 adverse events, CRS, or ICANS because fewer than 10 reports were available for each outcome. Given the limited number of studies and the sparse‐event nature of several analyses, the absence of statistically significant Egger's test results should not be interpreted as definitive evidence of the absence of publication bias (Table 3).

**TABLE 3:** Egger's test results.

Outcome	*k*	Egger β1 (SE)	*z*	*p*
Overall response (OR)	12	−0.793 (1.472)	−0.539	0.590
Complete response (CR)	12	−2.720 (1.620)	−1.679	0.093
Partial response (PR)	12	−1.250 (1.389)	−0.900	0.368
Stable disease (SD)	12	−1.309 (0.866)	−1.510	0.131
Progressive disease (PD)	13	0.250 (1.033)	0.242	0.809

## 4 Discussion

The present study systematically reviewed methodologies and evaluated the clinical effectiveness of CAR‐T‐cell‐based immunotherapies in patients with glioma through a systematic review and meta‐analysis. The included evidence was derived predominantly from early‐phase studies, most of which enrolled patients with recurrent or progressive glioblastoma, while a smaller number involved paediatric diffuse midline glioma or mixed glioma populations. The marked heterogeneity in patient characteristics, target antigens, CAR designs and routes of administration likely contributed to the variability observed across outcomes (Table 1).

The pooled efficacy results indicate that CAR‐T‐cell therapy produced objective responses in a subset of patients, with an OR rate of 16.9% (Figure 2) and a CR rate of 8.1% (Table 2). SD was more common than objective response, whereas PD remained frequent across studies (Figures 4 and 5). These findings suggest that CAR‐T‐cell therapy has genuine antitumor activity in glioma; however, its clinical impact is still constrained by major biological barriers, including antigen heterogeneity, the immunosuppressive tumour microenvironment and limited trafficking and persistence of effector cells within the central nervous system (Figures 2, 3, 4, 5).

Exploratory subgroup analyses suggested possible differences in efficacy according to target antigen. GD2‐ and IL‐13Rα2‐targeted CAR‐T‐cell therapies showed comparatively higher pooled objective response estimates, whereas the EGFRvIII subgroup was associated with a high proportion of PD. These patterns may reflect differences in antigen expression, immune escape, or CAR‐T‐cell activity in the glioma microenvironment. However, because most subgroup estimates were based on only one or two studies, these findings should be interpreted cautiously and regarded as hypothesis‐generating rather than definitive comparative evidence (Table 2).

The safety profile appeared acceptable overall, with grade ≥ 3 adverse events reported in a minority of patients (Figure 6). CRS and ICANS were observed across several studies, although their reported frequencies varied substantially (Figures 7 and 8). This variability likely reflects differences in CAR construct, treatment route, dose intensity, and the use of lymphodepleting chemotherapy or combination strategies (Figures 6, 7, 8). Importantly, the toxicity burden appears manageable in the context of heavily pretreated glioma populations, supporting the feasibility of continued clinical development (Table 1).

The robustness of these findings was supported by sensitivity analyses, which showed that no single study materially changed the overall pooled estimates (Table S3; Figures [Link], [Link], [Link], [Link], [Link], [Link], [Link], [Link]). Although some individual reports contributed to heterogeneity in CRS and ICANS, the overall conclusions remained stable after leave‐one‐out analysis (Table S3; Figures [Link], [Link], [Link], [Link], [Link], [Link], [Link], [Link]). In addition, no strong evidence of publication bias was detected for the main efficacy outcomes, and the funnel plot for OR did not suggest pronounced asymmetry. Nevertheless, these findings should be interpreted with caution given the limited number of studies and the sparse‐event structure of several outcomes (Figure 9).

Jang et al. [20] performed a systematic review and meta‐analysis to assess the safety and efficacy of CAR‐T‐cell therapy in glioma. Their search of PubMed, Embase and the Cochrane Library captured studies published up to 30 June 2021. Toxicity data were summarised, and pooled estimates of OR and OS were calculated using a random‐effects model with inverse‐variance weighting and a quantile estimation method, respectively. Eight studies were included, comprising 63 patients with recurrent glioma treated with various CAR‐T‐cell regimens. CRS of grade ≤ 2 occurred in six patients (9.5%), and non‐critical neurological adverse events were reported in 16 (25.4%). The pooled OR was 5.1% (95% CI, 0.0–10.4; *I* ^2^ = 0.05%), and the median OS was 8.1 months (95% CI, 6.7–9.5; *I* ^2^ = 0.00%). The authors concluded that, while CAR‐T‐cell therapy appears relatively safe in this population, its clinical efficacy remains limited and warrants further investigation [20].

In another systematic review, Agosti et al. [21] examined molecular targets and therapeutic strategies for CAR‐T‐cell therapy in glioma. Their search of PubMed, Web of Science and Scopus (conducted on 9 May 2024) yielded eight eligible articles published between 2015 and 2023, representing 151 patients. The studies differed in CAR construct and administration route; EGFRvIII‐targeted CAR‐T cells were the most frequently studied (three studies, 37.5%), and intravenous injection was the predominant route (62.5%). Reported median OS values ranged from 5.5 to 11.1 months. Progression‐free survival was reported in only two studies (7.5 months and 1.3 months). The reviewers emphasised the therapeutic potential of targeting antigens such as EGFRvIII and IL13Rα2, but noted persistent challenges including antigen escape, intratumoral heterogeneity and the immunosuppressive tumour microenvironment. They highlighted the need for optimised delivery methods, combination regimens and personalised approaches to improve clinical outcomes [21].

Azzam et al. [22] conducted a meta‐analysis of phase I trials to evaluate the safety and efficacy outcomes of CAR‐T‐cell therapy in relapsed glioma, incorporating eight studies with a total of 108 patients. Endpoints included OS, complete response rates, PD and treatment‐related adverse events, including CRS. The pooled median OS was 6.49 months, which did not differ substantially from historical benchmarks. Complete responses were uncommon, and a substantial proportion of patients experienced SD (44%) or PD (58%) following therapy. Notably, CAR‐T‐related encephalopathy was reported in 37% of patients, whereas CRS was infrequent (3%). The authors described their analytic approach as a novel predictive technique and cautioned that the findings were more hypothesis‐generating than confirmatory. They recommended additional clinical trials and methodological refinements to enhance both efficacy and safety [22].

Finally, a 2025 review by Czyżewski et al. provided a comprehensive synthesis of recent advances, ongoing challenges and future directions for CAR‐T‐cell therapy in glioma, including a tabulated summary of relevant studies (see table 1 in reference [16]).

This systematic review and meta‐analysis has both important strengths and limitations. Key strengths include the comprehensive inclusion of 306 patients across 23 studies—nearly double the sample size of prior reviews—updated evidence through June 2026, rigorous quality assessment using the JBI Critical Appraisal Checklist, extensive sensitivity analyses and the first systematic antigen‐specific subgroup analysis in this field. Our prospectively registered protocol and adherence to PRISMA 2020 guidelines (Table S4) ensure methodological transparency.

However, several limitations warrant consideration. The included evidence consists predominantly of early‐phase, single‐arm trials with inherent heterogeneity in CAR designs, delivery routes and patient populations. While this reflects the evolving nature of the field, it limits definitive comparative effectiveness conclusions. Our broad keyword‐based search strategy prioritised sensitivity over precision; incorporation of controlled vocabulary and field‐specific restrictions in future reviews may enhance efficiency. The 1992 start date, though aligned with CAR‐T development, represents an arbitrary restriction that could be eliminated in future updates. Finally, sparse events in some analyses and limited study numbers for certain outcomes constrain the precision of specific estimates and publication bias assessment.

On balance, despite these limitations, this review represents the most comprehensive and current synthesis available, providing actionable insights for trial design, target selection and patient counselling while identifying critical gaps for future research.

## 5 Conclusions and Future Perspective

The synthesis of efficacy outcomes and methodological limitations from prior glioma immunotherapy studies facilitates the identification of novel therapeutic strategies and elucidates mechanisms of treatment failure, thereby informing approaches to overcome resistance. Overall, the current meta‐analysis indicates that CAR‐T‐cell therapy has shown early promise in glioma; however, its efficacy remains inconsistent and incomplete. Future progress will depend on improved antigen selection, multi‐target CAR designs, enhanced locoregional delivery and rational combination strategies to overcome the immunosuppressive tumour microenvironment. Larger, well‐designed trials with standardised reporting are needed to determine which approaches can achieve durable clinical benefit. Future investigations should prioritise multicentre trials with harmonised eligibility criteria and standardised outcomes, incorporate subgroup analyses and meta‐analytic frameworks to explore heterogeneity, and ensure longitudinal follow‐up for both efficacy and late toxicities. Mechanistic studies should evaluate combination strategies, optimise CAR constructs and identify predictive biomarkers for patient selection. Collectively, these measures will reduce uncertainty and better define the therapeutic role of CAR‐T cells in glioma.

## Author Contributions

**Seyed Mostafa Mostafavi Zadeh:** conceptualization, investigation, writing – original draft, data curation, supervision, software, formal analysis, methodology, validation, visualization, writing – review and editing. **Sima Kalantari:** conceptualization, writing – original draft, writing – review and editing, methodology, validation, visualization, data curation, supervision, software, formal analysis, investigation. **Shaghayegh Shafiee Dastgerdi:** conceptualization, writing – original draft, writing – review and editing, investigation, data curation, supervision. **Hossein Sharifi:** conceptualization, writing – original draft, writing – review and editing, methodology, validation, visualization, investigation, data curation, supervision. **Mahdi Mohammadi:** investigation, validation, visualization, writing – review and editing, formal analysis, software, data curation. **Raheleh Roudi:** conceptualization, writing – original draft, writing – review and editing, investigation, methodology, validation, visualization, data curation, supervision. **Ahmad Shariftabrizi:** writing – original draft, writing – review and editing, supervision. **Abolfazl Kalantari:** writing – original draft, writing – review and editing, supervision. **Davoud Ahmadvand:** writing – original draft, writing – review and editing, conceptualization, investigation, methodology, validation, data curation, supervision, project administration. **Maryam Yazdi:** writing – original draft, writing – review and editing. **Reza Nedaeinia:** conceptualization, writing – original draft, writing – review and editing, supervision, methodology, investigation.

## Funding

This work was supported by Iran University of Medical Sciences, Tehran, Iran (Grant Code: IR.IUMS.REC.1402.3.75.25755). The funding institution had no involvement in the research design, data acquisition, analysis, interpretation, or the decision to publish.

## Ethics Statement

This systematic review and meta‐analysis was approved by the Ethics Committee of Iran University of Medical Sciences, Tehran, Iran (Ethics Code: IR.IUMS.REC.1402.1227). As this study involved analysis of previously published data, informed consent was not required.

## Conflicts of Interest

The authors declare no conflicts of interest.

## Supporting information


**Figure S1:** Leave‐one‐out sensitivity analysis for overall response. This figure displays the pooled overall response proportion and 95% confidence interval after sequential omission of each individual study.

**Figure S2:** Leave‐one‐out sensitivity analysis for complete response. This figure displays the pooled complete response proportion and 95% confidence interval after sequential omission of each individual study.

**Figure S3:** Leave‐one‐out sensitivity analysis for partial response. This figure displays the pooled partial response proportion and 95% confidence interval after sequential omission of each individual study.

**Figure S4:** Leave‐one‐out sensitivity analysis for stable disease. This figure displays the pooled stable disease proportion and 95% confidence interval after sequential omission of each individual study.

**Figure S5:** Leave‐one‐out sensitivity analysis for progressive disease. This figure displays the pooled progressive disease proportion and 95% confidence interval after sequential omission of each individual study.

**Figure S6:** Leave‐one‐out sensitivity analysis for grade ≥ 3 adverse events. This figure displays the pooled grade ≥ 3 adverse event proportion and 95% confidence interval after sequential omission of each individual study.

**Figure S7:** Leave‐one‐out sensitivity analysis for cytokine release syndrome. This figure displays the pooled cytokine release syndrome proportion and 95% confidence interval after sequential omission of each individual study.

**Figure S8:** Leave‐one‐out sensitivity analysis for immune effector cell‐associated neurotoxicity syndrome. This figure displays the pooled immune effector cell‐associated neurotoxicity syndrome proportion and 95% confidence interval after sequential omission of each individual study.

**Table S1:** Database search strategies. This table provides the complete, database‐specific search strategies used for PubMed/MEDLINE, Embase, Scopus, Web of Science and ProQuest, including all Medical Subject Headings (MeSH) terms, Emtree terms, keywords and Boolean operators employed in the systematic literature search.

**Table S2:** Joanna Briggs Institute (JBI) Critical Appraisal Checklist results for included studies. This table presents detailed item‐level quality assessment results for all 23 included studies across 10 methodological domains, including clarity of inclusion criteria, reliability of condition ascertainment, consecutive inclusion, demographic reporting, outcome reporting, follow‐up, study setting and statistical analysis.

**Table S3:** Leave‐one‐out sensitivity analysis results for all outcomes. This table provides numerical results of sensitivity analyses showing the impact of sequentially omitting each individual study on pooled effect estimates for overall response, complete response, partial response, stable disease, progressive disease, grade ≥ 3 adverse events, cytokine release syndrome and immune effector cell‐associated neurotoxicity syndrome.

**Table S4:** PRISMA 2020 Checklist demonstrating reporting compliance and location of items in the manuscript.

## Data Availability

All relevant datasets generated and analysed during this study are available from the corresponding author upon reasonable request.
