# Supplementary material for: Efficacy of CAR‐T‐Cell‐Based Immunotherapies in Patients With Glioma: A Systematic Review and Meta‐Analysis
Source: J Cell Mol Med. 2026 Sep 25;30(18):e71358. doi: 10.1111/jcmm.71358 (PMC13614879; doi:10.1111/jcmm.71358)
Supplement: Supplementary file 1 [file JCMM-30-e71358-s004.docx]

| **PubMed/MEDLINE**  Supplementary Table S1. Database search strategies. This table provides the complete, database-specific search strategies used for PubMed/MEDLINE, Embase, Scopus, Web of Science, and ProQuest, including all Medical Subject Headings (MeSH) terms, Emtree terms, keywords, and Boolean operators employed in the systematic literature search. | Number of papers |
| --- | --- |
| ((Glioma*[ALL] OR "Glial Cell Tumor*"[ALL] OR (Tumor*[ALL] AND "Glial Cell"[ALL]) OR "Mixed Glioma"[ALL] OR (Glioma*[ALL] AND Mixed[ALL]) OR "Mixed Glioma*"[ALL] OR "Malignant Glioma*"[ALL] OR (Glioma*[ALL] AND Malignant[ALL]) OR glioblastoma[ALL] OR "anaplastic astrocytoma"[ALL] OR "diffuse astrocytoma"[ALL] OR "anaplastic oligodendroglioma"[ALL] OR oligodendroglioma[ALL])  AND  ((Immunotherapy[ALL] AND "Adoptive Cellular"[ALL]) OR ("Adoptive Immunotherapy"[ALL]) OR ("Adoptive Immunotherapies"[ALL]) OR (Immunotherapies[ALL] AND Adoptive[ALL]) OR ("Cellular Immunotherapy"[ALL] AND Adoptive[ALL]) OR ("Cellular Immunotherapies"[ALL] AND Adoptive[ALL]) OR (Immunotherapies[ALL] AND "Adoptive Cellular"[ALL]) OR "Adoptive Cellular Immunotherapies"[ALL] OR "Adoptive Cellular Immunotherapy"[ALL] OR "Chimeric Antigen Receptor Therapy"[ALL] OR "Chimeric Antigen Receptor Therapy"[ALL] OR "CAR T-Cell Therapy"[ALL] OR "CAR T-Cell Therapies"[ALL] OR "CAR T Cell Therapy"[ALL] OR ("T-Cell Therapies"[ALL] AND CAR[ALL]) OR ("T-Cell Therapy"[ALL] AND CAR[ALL]) OR (Therapies[ALL] AND "CAR T-Cell"[ALL]) OR (Therapy[ALL] AND "CAR T-Cell"[ALL]))  AND  (1992/01/01:2026/06/03[dp])) | 1038 |
| **Embase** |  |
| ((Glioma* OR ‘Glial Cell Tumor*’ OR (Tumor* AND ‘Glial Cell’) OR ‘Mixed Glioma’ OR (Glioma* AND Mixed) OR ‘Mixed Glioma*’ OR ‘Malignant Glioma*’ OR (Glioma* AND Malignant) OR glioblastoma OR ‘anaplastic astrocytoma’ OR ‘diffuse astrocytoma’ OR ‘anaplastic oligodendroglioma’ OR oligodendroglioma)  AND  ((Immunotherapy AND ‘Adoptive Cellular’) OR (‘Adoptive Immunotherapy’) OR (‘Adoptive Immunotherapies’) OR (Immunotherapies AND Adoptive) OR (‘Cellular Immunotherapy’ AND Adoptive) OR (‘Cellular Immunotherapies’ AND Adoptive) OR (Immunotherapies AND ‘Adoptive Cellular’) OR ‘Adoptive Cellular Immunotherapies’ OR ‘Adoptive Cellular Immunotherapy’ OR ‘Chimeric Antigen Receptor Therapy’ OR ‘Chimeric Antigen Receptor Therapy’ OR ‘CAR T-Cell Therapy’ OR ‘CAR T-Cell Therapies’ OR ‘CAR T Cell Therapy’ OR (‘T-Cell Therapies’ AND CAR) OR (‘T-Cell Therapy’ AND CAR) OR (Therapies AND ‘CAR T-Cell’) OR (Therapy AND ‘CAR T-Cell’))  AND  [1992-2026]/PY) | 1971 |
| **Scopus** |  |
| ((TITLE-ABS-KEY(Glioma*) OR TITLE-ABS-KEY(“Glial Cell Tumor*”) OR TITLE-ABS-KEY(Tumor* AND “Glial Cell”) OR TITLE-ABS-KEY(“Mixed Glioma”) OR TITLE-ABS-KEY(Glioma* AND Mixed) OR TITLE-ABS-KEY(“Mixed Glioma*”) OR TITLE-ABS-KEY(“Malignant Glioma*”) OR TITLE-ABS-KEY(Glioma* AND Malignant) OR TITLE-ABS-KEY(glioblastoma) OR TITLE-ABS-KEY(“anaplastic astrocytoma”) OR TITLE-ABS-KEY(“diffuse astrocytoma”) OR TITLE-ABS-KEY(“anaplastic oligodendroglioma”) OR TITLE-ABS-KEY(oligodendroglioma))  AND  (TITLE-ABS-KEY(Immunotherapy AND “Adoptive Cellular”) OR TITLE-ABS-KEY(“Adoptive Immunotherapy”) OR TITLE-ABS-KEY(“Adoptive Immunotherapies”) OR TITLE-ABS-KEY(Immunotherapies AND Adoptive) OR TITLE-ABS-KEY(“Cellular Immunotherapy” AND Adoptive) OR TITLE-ABS-KEY(“Cellular Immunotherapies” AND Adoptive) OR TITLE-ABS-KEY(Immunotherapies AND “Adoptive Cellular”) OR TITLE-ABS-KEY(“Adoptive Cellular Immunotherapies”) OR TITLE-ABS-KEY(“Adoptive Cellular Immunotherapy”) OR TITLE-ABS-KEY(“Chimeric Antigen Receptor Therapy”) OR ALL(“Chimeric Antigen Receptor Therapy”) OR TITLE-ABS-KEY(“CAR T-Cell Therapy”) OR ALL(“CAR T-Cell Therapies”) OR TITLE-ABS-KEY(“CAR T Cell Therapy”) OR TITLE-ABS-KEY(“T-Cell Therapies” AND CAR) OR TITLE-ABS-KEY(“T-Cell Therapy” AND CAR) OR TITLE-ABS-KEY(Therapies AND “CAR T-Cell”) OR TITLE-ABS-KEY(Therapy AND “CAR T-Cell”))  AND  PUBYEAR > 1991 AND PUBYEAR<2027) | 3120 |
| **WOS** |  |
| ((ALL=(Glioma*) OR ALL=(‘Glial Cell Tumor*’) OR ALL=(Tumor* AND ‘Glial Cell’) OR ALL=(‘Mixed Glioma’) OR ALL=(Glioma* AND Mixed) OR ALL=(‘Mixed Glioma*’) OR ALL=(‘Malignant Glioma*’) OR ALL=(Glioma* AND Malignant) OR ALL=(glioblastoma) OR ALL=(‘anaplastic astrocytoma’) OR ALL=(‘diffuse astrocytoma’) OR ALL=(‘anaplastic oligodendroglioma’) OR ALL=(oligodendroglioma))  AND  (ALL=(Immunotherapy AND ‘Adoptive Cellular’) OR ALL=(‘Adoptive Immunotherapy’) OR ALL=(‘Adoptive Immunotherapies’) OR ALL=(Immunotherapies AND Adoptive) OR ALL=(‘Cellular Immunotherapy’ AND Adoptive) OR ALL=(‘Cellular Immunotherapies’ AND Adoptive) OR ALL=(Immunotherapies AND ‘Adoptive Cellular’) OR ALL=(‘Adoptive Cellular Immunotherapies’) OR ALL=(‘Adoptive Cellular Immunotherapy’) OR ALL=(‘Chimeric Antigen Receptor Therapy’) OR ALL=(‘Chimeric Antigen Receptor Therapy’) OR ALL=(‘CAR T-Cell Therapy’) OR ALL=(‘CAR T-Cell Therapies’) OR ALL=(‘CAR T Cell Therapy’) OR ALL=(‘T-Cell Therapies’ AND CAR) OR ALL=(‘T-Cell Therapy’ AND CAR) OR ALL=(Therapies AND ‘CAR T-Cell’) OR ALL=(Therapy AND ‘CAR T-Cell’))  AND  PY=(1992-2026)) | 1,534 |
| **ProQuest** |  |
| (AB,TI(Glioma*) OR AB,TI(‘Glial Cell Tumor*’) OR AB,TI(Tumor* AND ‘Glial Cell’) OR AB,TI(‘Mixed Glioma’) OR AB,TI(Glioma* AND Mixed) OR AB,TI(‘Mixed Glioma*’) OR AB,TI(‘Malignant Glioma*’) OR AB,TI(Glioma* AND Malignant) OR AB,TI(glioblastoma) OR AB,TI(‘anaplastic astrocytoma’) OR AB,TI(‘diffuse astrocytoma’) OR AB,TI(‘anaplastic oligodendroglioma’) OR AB,TI(oligodendroglioma))  AND  (AB,TI(Immunotherapy AND ‘Adoptive Cellular’) OR AB,TI(‘Adoptive Immunotherapy’) OR AB,TI(‘Adoptive Immunotherapies’) OR AB,TI(Immunotherapies AND Adoptive) OR AB,TI(‘Cellular Immunotherapy’ AND Adoptive) OR AB,TI(‘Cellular Immunotherapies’ AND Adoptive) OR AB,TI(Immunotherapies AND ‘Adoptive Cellular’) OR AB,TI(‘Adoptive Cellular Immunotherapies’) OR AB,TI(‘Adoptive Cellular Immunotherapy’) OR AB,TI(‘Chimeric Antigen Receptor Therapy’) OR AB,TI(‘Chimeric Antigen Receptor Therapy’) OR AB,TI(‘CAR T-Cell Therapy’) OR AB,TI(‘CAR T-Cell Therapies’) OR AB,TI(‘CAR T Cell Therapy’) OR AB,TI(‘T-Cell Therapies’ AND CAR) OR AB,TI(‘T-Cell Therapy’ AND CAR) OR AB,TI(Therapies AND ‘CAR T-Cell’) OR AB,TI(Therapy AND ‘CAR T-Cell’))  AND  YR(19920101-20260603) | 60 |

**Raw syntax**

((Glioma* OR “Glial Cell Tumor*” OR (Tumor* AND “Glial Cell”) OR “Mixed Glioma” OR (Glioma* AND Mixed) OR “Mixed Glioma*” OR “Malignant Glioma*” OR (Glioma* AND Malignant) OR glioblastoma OR “anaplastic astrocytoma” OR “diffuse astrocytoma” OR “anaplastic oligodendroglioma” OR oligodendroglioma)

AND

((Immunotherapy AND “Adoptive Cellular”) OR (“Adoptive Immunotherapy”) OR (“Adoptive Immunotherapies”) OR (Immunotherapies AND Adoptive) OR (“Cellular Immunotherapy” AND Adoptive) OR (“Cellular Immunotherapies” AND Adoptive) OR (Immunotherapies AND “Adoptive Cellular”) OR “Adoptive Cellular Immunotherapies” OR “Adoptive Cellular Immunotherapy” OR “Chimeric Antigen Receptor Therapy” OR “Chimeric Antigen Receptor Therapy” OR “CAR T-Cell Therapy” OR “CAR T-Cell Therapies” OR “CAR T Cell Therapy” OR (“T-Cell Therapies” AND CAR) OR (“T-Cell Therapy” AND CAR) OR (Therapies AND “CAR T-Cell”) OR (Therapy AND “CAR T-Cell”))

AND

(1992/01/01:2026/06/03[dp]))
