# Supplementary material for: Efficacy of CAR‐T‐Cell‐Based Immunotherapies in Patients With Glioma: A Systematic Review and Meta‐Analysis
Source: J Cell Mol Med. 2026 Sep 25;30(18):e71358. doi: 10.1111/jcmm.71358 (PMC13614879; doi:10.1111/jcmm.71358)
Supplement: Supplementary file 2 [file JCMM-30-e71358-s008.docx]

| Supplementary Table S3. Leave-one-out sensitivity analysis results for all outcomes. This table provides numerical results of sensitivity analyses showing the impact of sequentially omitting each individual study on pooled effect estimates for overall response, complete response, partial response, stable disease, progressive disease, grade ≥3 adverse events, cytokine release syndrome, and immune effector cell-associated neurotoxicity syndrome. | | | | | | | | | | | | | | | | | | | |
| --- | --- | --- | --- | --- | --- | --- | --- | --- | --- | --- | --- | --- | --- | --- | --- | --- | --- | --- | --- |
| Outcome | **Omitted study** | **Omitted events** | **Omitted total** | **Remaining reports** | **Remaining outcome-evaluable participants** | **Pooled proportion** | **Lower 95% CI** | **Upper 95% CI** | **Change from full estimate (pp)** | **Absolute change from full estimate (pp)** | **tau²** | **I² (%)** | **Q** | **H²** | **z** | **P value** | **Full pooled proportion** | **Full lower 95% CI** | **Full upper 95% CI** |
| Overall response (OR) | Ahmed N 2017 | 1 | 16 | 11 | 137 | 18.3% | 8.3% | 35.7% | 1.5 | 1.5 | 1.239 | 58.3 | 23.968 | 2.397 | -3.237 | 0.001 | 16.9% | 8.0% | 32.2% |
|  | Brown CE 2024 | 3 | 58 | 11 | 95 | 19.8% | 9.3% | 37.3% | 2.9 | 2.9 | 1.025 | 47.1 | 18.914 | 1.891 | -3.115 | 0.002 | 16.9% | 8.0% | 32.2% |
|  | Lin FY 2024 | 2 | 11 | 11 | 142 | 16.6% | 7.2% | 34.0% | -0.2 | 0.2 | 1.409 | 60.4 | 25.226 | 2.523 | -3.328 | 0.001 | 16.9% | 8.0% | 32.2% |
|  | Lin Q 2021 | 0 | 3 | 11 | 150 | 17.1% | 7.7% | 33.7% | 0.3 | 0.3 | 1.293 | 60.3 | 25.179 | 2.518 | -3.428 | 0.001 | 16.9% | 8.0% | 32.2% |
|  | Bagley SJ 2024 (EGFRvIII cohort) | 0 | 7 | 11 | 146 | 17.8% | 8.2% | 34.6% | 1.0 | 1.0 | 1.248 | 59.4 | 24.626 | 2.463 | -3.353 | 0.001 | 16.9% | 8.0% | 32.2% |
|  | Gu J 2025 | 3 | 6 | 11 | 147 | 14.5% | 6.6% | 28.9% | -2.4 | 2.4 | 1.059 | 52.4 | 20.992 | 2.099 | -3.976 | 0.000 | 16.9% | 8.0% | 32.2% |
|  | Gust J 2025 | 0 | 4 | 11 | 149 | 17.3% | 7.9% | 34.0% | 0.5 | 0.5 | 1.284 | 60.1 | 25.064 | 2.506 | -3.399 | 0.001 | 16.9% | 8.0% | 32.2% |
|  | Liu Z 2023 | 4 | 8 | 11 | 145 | 14.2% | 6.5% | 28.2% | -2.7 | 2.7 | 0.983 | 48.3 | 19.328 | 1.933 | -4.079 | 0.000 | 16.9% | 8.0% | 32.2% |
|  | Barish ME 2025 | 0 | 4 | 11 | 149 | 17.3% | 7.9% | 34.0% | 0.5 | 0.5 | 1.284 | 60.1 | 25.064 | 2.506 | -3.399 | 0.001 | 16.9% | 8.0% | 32.2% |
|  | Vitanza NA 2025 | 1 | 18 | 11 | 135 | 18.5% | 8.5% | 35.8% | 1.7 | 1.7 | 1.210 | 57.7 | 23.656 | 2.366 | -3.230 | 0.001 | 16.9% | 8.0% | 32.2% |
|  | Bagley SJ 2025 (bivalent CAR) | 1 | 13 | 11 | 140 | 18.0% | 8.1% | 35.4% | 1.1 | 1.1 | 1.282 | 59.1 | 24.436 | 2.444 | -3.253 | 0.001 | 16.9% | 8.0% | 32.2% |
|  | Li X 2026 | 4 | 5 | 11 | 148 | 13.9% | 6.8% | 26.3% | -3.0 | 3.0 | 0.771 | 43.6 | 17.724 | 1.772 | -4.498 | 0.000 | 16.9% | 8.0% | 32.2% |
| Complete response (CR) | Ahmed N 2017 | 0 | 16 | 11 | 137 | 8.8% | 3.6% | 19.7% | 0.6 | 0.6 | 0.830 | 25.6 | 13.434 | 1.343 | -4.903 | 0.000 | 8.1% | 3.5% | 17.8% |
|  | Brown CE 2024 | 1 | 58 | 11 | 95 | 10.1% | 4.4% | 21.9% | 2.0 | 2.0 | 0.581 | 6.3 | 10.677 | 1.068 | -4.708 | 0.000 | 8.1% | 3.5% | 17.8% |
|  | Lin FY 2024 | 0 | 11 | 11 | 142 | 8.5% | 3.5% | 19.3% | 0.4 | 0.4 | 0.865 | 27.7 | 13.828 | 1.383 | -4.935 | 0.000 | 8.1% | 3.5% | 17.8% |
|  | Lin Q 2021 | 0 | 3 | 11 | 150 | 7.8% | 3.2% | 17.9% | -0.3 | 0.3 | 0.895 | 29.4 | 14.155 | 1.415 | -5.110 | 0.000 | 8.1% | 3.5% | 17.8% |
|  | Bagley SJ 2024 (EGFRvIII cohort) | 0 | 7 | 11 | 146 | 8.2% | 3.4% | 18.8% | 0.1 | 0.1 | 0.890 | 29.1 | 14.112 | 1.411 | -4.986 | 0.000 | 8.1% | 3.5% | 17.8% |
|  | Gu J 2025 | 3 | 6 | 11 | 147 | 5.5% | 2.5% | 11.4% | -2.7 | 2.7 | 0.000 | 0.0 | 4.483 | 0.448 | -6.970 | 0.000 | 8.1% | 3.5% | 17.8% |
|  | Gust J 2025 | 0 | 4 | 11 | 149 | 7.9% | 3.2% | 18.2% | -0.2 | 0.2 | 0.899 | 29.6 | 14.202 | 1.420 | -5.065 | 0.000 | 8.1% | 3.5% | 17.8% |
|  | Liu Z 2023 | 0 | 8 | 11 | 145 | 8.3% | 3.4% | 18.9% | 0.2 | 0.2 | 0.885 | 28.8 | 14.049 | 1.405 | -4.970 | 0.000 | 8.1% | 3.5% | 17.8% |
|  | Barish ME 2025 | 0 | 4 | 11 | 149 | 7.9% | 3.2% | 18.2% | -0.2 | 0.2 | 0.899 | 29.6 | 14.202 | 1.420 | -5.065 | 0.000 | 8.1% | 3.5% | 17.8% |
|  | Vitanza NA 2025 | 0 | 18 | 11 | 135 | 8.8% | 3.7% | 19.8% | 0.7 | 0.7 | 0.816 | 24.7 | 13.279 | 1.328 | -4.895 | 0.000 | 8.1% | 3.5% | 17.8% |
|  | Bagley SJ 2025 (bivalent CAR) | 0 | 13 | 11 | 140 | 8.6% | 3.6% | 19.5% | 0.5 | 0.5 | 0.851 | 26.8 | 13.670 | 1.367 | -4.919 | 0.000 | 8.1% | 3.5% | 17.8% |
|  | Li X 2026 | 1 | 5 | 11 | 148 | 7.2% | 2.9% | 17.0% | -0.9 | 0.9 | 0.879 | 25.9 | 13.495 | 1.350 | -5.199 | 0.000 | 8.1% | 3.5% | 17.8% |
| Partial response (PR) | Ahmed N 2017 | 1 | 16 | 11 | 137 | 14.0% | 6.4% | 28.0% | 0.9 | 0.9 | 0.989 | 47.8 | 19.159 | 1.916 | -4.076 | 0.000 | 13.1% | 6.3% | 25.5% |
|  | Brown CE 2024 | 2 | 58 | 11 | 95 | 15.9% | 7.7% | 30.1% | 2.8 | 2.8 | 0.695 | 31.2 | 14.534 | 1.453 | -3.965 | 0.000 | 13.1% | 6.3% | 25.5% |
|  | Lin FY 2024 | 2 | 11 | 11 | 142 | 12.5% | 5.5% | 26.1% | -0.7 | 0.7 | 1.083 | 49.6 | 19.860 | 1.986 | -4.219 | 0.000 | 13.1% | 6.3% | 25.5% |
|  | Lin Q 2021 | 0 | 3 | 11 | 150 | 13.1% | 6.0% | 26.3% | 0.0 | 0.0 | 1.003 | 50.0 | 20.005 | 2.001 | -4.308 | 0.000 | 13.1% | 6.3% | 25.5% |
|  | Bagley SJ 2024 (EGFRvIII cohort) | 0 | 7 | 11 | 146 | 13.7% | 6.3% | 27.2% | 0.5 | 0.5 | 0.977 | 49.0 | 19.606 | 1.961 | -4.219 | 0.000 | 13.1% | 6.3% | 25.5% |
|  | Gu J 2025 | 0 | 6 | 11 | 147 | 13.6% | 6.2% | 27.0% | 0.4 | 0.4 | 0.986 | 49.3 | 19.727 | 1.973 | -4.233 | 0.000 | 13.1% | 6.3% | 25.5% |
|  | Gust J 2025 | 0 | 4 | 11 | 149 | 13.3% | 6.1% | 26.6% | 0.2 | 0.2 | 0.999 | 49.8 | 19.939 | 1.994 | -4.275 | 0.000 | 13.1% | 6.3% | 25.5% |
|  | Liu Z 2023 | 4 | 8 | 11 | 145 | 10.4% | 5.1% | 20.2% | -2.7 | 2.7 | 0.512 | 18.7 | 12.300 | 1.230 | -5.414 | 0.000 | 13.1% | 6.3% | 25.5% |
|  | Barish ME 2025 | 0 | 4 | 11 | 149 | 13.3% | 6.1% | 26.6% | 0.2 | 0.2 | 0.999 | 49.8 | 19.939 | 1.994 | -4.275 | 0.000 | 13.1% | 6.3% | 25.5% |
|  | Vitanza NA 2025 | 1 | 18 | 11 | 135 | 14.2% | 6.5% | 28.2% | 1.0 | 1.0 | 0.969 | 47.1 | 18.901 | 1.890 | -4.066 | 0.000 | 13.1% | 6.3% | 25.5% |
|  | Bagley SJ 2025 (bivalent CAR) | 1 | 13 | 11 | 140 | 13.7% | 6.2% | 27.7% | 0.6 | 0.6 | 1.018 | 48.8 | 19.534 | 1.953 | -4.101 | 0.000 | 13.1% | 6.3% | 25.5% |
|  | Li X 2026 | 3 | 5 | 11 | 148 | 10.7% | 5.2% | 20.8% | -2.5 | 2.5 | 0.604 | 26.3 | 13.576 | 1.358 | -5.285 | 0.000 | 13.1% | 6.3% | 25.5% |
| Stable disease (SD) | Ahmed N 2017 | 7 | 16 | 11 | 137 | 46.0% | 31.3% | 61.5% | -0.6 | 0.6 | 0.448 | 44.6 | 18.055 | 1.806 | -0.500 | 0.617 | 46.6% | 34.3% | 59.3% |
|  | Brown CE 2024 | 26 | 58 | 11 | 95 | 45.6% | 30.2% | 61.9% | -1.0 | 1.0 | 0.511 | 44.1 | 17.896 | 1.790 | -0.521 | 0.602 | 46.6% | 34.3% | 59.3% |
|  | Lin FY 2024 | 5 | 11 | 11 | 142 | 45.8% | 31.4% | 61.0% | -0.7 | 0.7 | 0.429 | 44.8 | 18.130 | 1.813 | -0.532 | 0.594 | 46.6% | 34.3% | 59.3% |
|  | Lin Q 2021 | 1 | 3 | 11 | 150 | 47.0% | 34.0% | 60.3% | 0.4 | 0.4 | 0.296 | 44.2 | 17.914 | 1.791 | -0.440 | 0.660 | 46.6% | 34.3% | 59.3% |
|  | Bagley SJ 2024 (EGFRvIII cohort) | 0 | 7 | 11 | 146 | 48.7% | 37.0% | 60.4% | 2.1 | 2.1 | 0.168 | 33.0 | 14.933 | 1.493 | -0.220 | 0.826 | 46.6% | 34.3% | 59.3% |
|  | Gu J 2025 | 3 | 6 | 11 | 147 | 45.6% | 31.8% | 60.2% | -0.9 | 0.9 | 0.386 | 44.8 | 18.127 | 1.813 | -0.583 | 0.560 | 46.6% | 34.3% | 59.3% |
|  | Gust J 2025 | 1 | 4 | 11 | 149 | 47.6% | 34.9% | 60.7% | 1.0 | 1.0 | 0.263 | 42.5 | 17.394 | 1.739 | -0.352 | 0.725 | 46.6% | 34.3% | 59.3% |
|  | Liu Z 2023 | 1 | 8 | 11 | 145 | 49.1% | 37.7% | 60.6% | 2.6 | 2.6 | 0.140 | 33.8 | 15.112 | 1.511 | -0.145 | 0.885 | 46.6% | 34.3% | 59.3% |
|  | Barish ME 2025 | 3 | 4 | 11 | 149 | 44.9% | 32.2% | 58.4% | -1.6 | 1.6 | 0.296 | 41.3 | 17.026 | 1.703 | -0.734 | 0.463 | 46.6% | 34.3% | 59.3% |
|  | Vitanza NA 2025 | 15 | 18 | 11 | 135 | 43.7% | 35.2% | 52.6% | -2.9 | 2.9 | 0.000 | 1.4 | 10.144 | 1.014 | -1.383 | 0.167 | 46.6% | 34.3% | 59.3% |
|  | Bagley SJ 2025 (bivalent CAR) | 8 | 13 | 11 | 140 | 44.0% | 30.4% | 58.7% | -2.5 | 2.5 | 0.360 | 41.2 | 17.005 | 1.701 | -0.795 | 0.427 | 46.6% | 34.3% | 59.3% |
|  | Li X 2026 | 1 | 5 | 11 | 148 | 48.2% | 35.8% | 60.8% | 1.6 | 1.6 | 0.226 | 40.5 | 16.808 | 1.681 | -0.282 | 0.778 | 46.6% | 34.3% | 59.3% |
| Progressive disease (PD) | Ahmed N 2017 | 8 | 16 | 12 | 154 | 43.4% | 25.0% | 63.8% | -0.5 | 0.5 | 1.257 | 60.5 | 27.881 | 2.535 | -0.626 | 0.531 | 43.9% | 27.7% | 61.5% |
|  | Brown CE 2024 | 29 | 58 | 12 | 112 | 43.3% | 24.6% | 64.1% | -0.6 | 0.6 | 1.295 | 59.3 | 27.035 | 2.458 | -0.623 | 0.533 | 43.9% | 27.7% | 61.5% |
|  | Goff S 2019 | 16 | 17 | 12 | 153 | 39.7% | 28.3% | 52.2% | -4.2 | 4.2 | 0.215 | 43.6 | 19.510 | 1.774 | -1.614 | 0.107 | 43.9% | 27.7% | 61.5% |
|  | Lin FY 2024 | 4 | 11 | 12 | 159 | 44.9% | 26.4% | 65.0% | 1.0 | 1.0 | 1.226 | 60.3 | 27.692 | 2.517 | -0.484 | 0.628 | 43.9% | 27.7% | 61.5% |
|  | Lin Q 2021 | 2 | 3 | 12 | 167 | 42.6% | 25.8% | 61.4% | -1.3 | 1.3 | 1.029 | 60.0 | 27.518 | 2.502 | -0.768 | 0.442 | 43.9% | 27.7% | 61.5% |
|  | Bagley SJ 2024 (EGFRvIII cohort) | 7 | 7 | 12 | 163 | 40.8% | 26.2% | 57.2% | -3.1 | 3.1 | 0.669 | 54.3 | 24.044 | 2.186 | -1.101 | 0.271 | 43.9% | 27.7% | 61.5% |
|  | Gu J 2025 | 0 | 6 | 12 | 164 | 46.4% | 29.9% | 63.7% | 2.5 | 2.5 | 0.838 | 56.7 | 25.413 | 2.310 | -0.403 | 0.687 | 43.9% | 27.7% | 61.5% |
|  | Gust J 2025 | 3 | 4 | 12 | 166 | 41.9% | 25.6% | 60.2% | -2.0 | 2.0 | 0.941 | 58.9 | 26.769 | 2.434 | -0.870 | 0.384 | 43.9% | 27.7% | 61.5% |
|  | Liu Z 2023 | 3 | 8 | 12 | 162 | 44.7% | 26.4% | 64.7% | 0.8 | 0.8 | 1.210 | 60.5 | 27.860 | 2.533 | -0.508 | 0.611 | 43.9% | 27.7% | 61.5% |
|  | Barish ME 2025 | 1 | 4 | 12 | 166 | 45.4% | 27.8% | 64.2% | 1.4 | 1.4 | 1.062 | 59.9 | 27.431 | 2.494 | -0.475 | 0.635 | 43.9% | 27.7% | 61.5% |
|  | Vitanza NA 2025 | 2 | 18 | 12 | 152 | 47.9% | 39.1% | 56.7% | 4.0 | 4.0 | 0.000 | 48.6 | 21.381 | 1.944 | -0.469 | 0.639 | 43.9% | 27.7% | 61.5% |
|  | Bagley SJ 2025 (bivalent CAR) | 4 | 13 | 12 | 157 | 45.6% | 27.1% | 65.4% | 1.7 | 1.7 | 1.175 | 59.1 | 26.911 | 2.446 | -0.423 | 0.672 | 43.9% | 27.7% | 61.5% |
|  | Li X 2026 | 0 | 5 | 12 | 165 | 46.2% | 29.6% | 63.8% | 2.3 | 2.3 | 0.871 | 57.4 | 25.803 | 2.346 | -0.415 | 0.678 | 43.9% | 27.7% | 61.5% |
| Grade ≥3 adverse events | Ahmed N 2017 | 3 | 17 | 8 | 52 | 18.0% | 8.0% | 35.8% | -0.6 | 0.6 | 0.476 | 7.8 | 7.596 | 1.085 | -3.189 | 0.001 | 18.6% | 9.6% | 33.1% |
|  | Brown CE 2022 | 0 | 6 | 8 | 63 | 19.8% | 10.0% | 35.3% | 1.2 | 1.2 | 0.289 | 0.0 | 6.996 | 0.999 | -3.462 | 0.001 | 18.6% | 9.6% | 33.1% |
|  | Lin FY 2024 | 1 | 11 | 8 | 58 | 20.3% | 10.1% | 36.4% | 1.6 | 1.6 | 0.289 | 0.0 | 6.805 | 0.972 | -3.301 | 0.001 | 18.6% | 9.6% | 33.1% |
|  | Lin Q 2021 | 0 | 3 | 8 | 66 | 18.9% | 9.4% | 34.4% | 0.3 | 0.3 | 0.330 | 7.3 | 7.553 | 1.079 | -3.533 | 0.000 | 18.6% | 9.6% | 33.1% |
|  | O'Rourke DM 2017 | 5 | 10 | 8 | 59 | 13.6% | 6.9% | 25.0% | -5.0 | **5.0** | 0.000 | 0.0 | 1.466 | 0.209 | -4.824 | 0.000 | 18.6% | 9.6% | 33.1% |
|  | Bagley SJ 2024 (EGFRvIII cohort) | 1 | 7 | 8 | 62 | 18.9% | 9.1% | 35.0% | 0.2 | 0.2 | 0.369 | 7.1 | 7.532 | 1.076 | -3.397 | 0.001 | 18.6% | 9.6% | 33.1% |
|  | Gu J 2025 | 0 | 6 | 8 | 63 | 19.8% | 10.0% | 35.3% | 1.2 | 1.2 | 0.289 | 0.0 | 6.996 | 0.999 | -3.462 | 0.001 | 18.6% | 9.6% | 33.1% |
|  | Gust J 2025 | 1 | 4 | 8 | 65 | 17.6% | 8.4% | 33.2% | -1.1 | 1.1 | 0.396 | 8.6 | 7.662 | 1.095 | -3.576 | 0.000 | 18.6% | 9.6% | 33.1% |
|  | Li X 2026 | 0 | 5 | 8 | 64 | 19.5% | 9.9% | 35.0% | 0.9 | 0.9 | 0.302 | 2.6 | 7.188 | 1.027 | -3.479 | 0.001 | 18.6% | 9.6% | 33.1% |
| Cytokine release syndrome (CRS) | Brown CE 2022 | 0 | 6 | 8 | 74 | 33.6% | 10.0% | 69.8% | 3.9 | 3.9 | 3.261 | 66.2 | 20.680 | 2.954 | -0.881 | 0.379 | 29.7% | 9.3% | 63.3% |
|  | Lin FY 2024 | 6 | 11 | 8 | 69 | 25.7% | 6.3% | 63.9% | -4.0 | 4.0 | 3.729 | 66.8 | 21.112 | 3.016 | -1.275 | 0.202 | 29.7% | 9.3% | 63.3% |
|  | Lin Q 2021 | 2 | 3 | 8 | 77 | 25.4% | 6.7% | 61.9% | -4.2 | 4.2 | 3.445 | 68.0 | 21.891 | 3.127 | -1.352 | 0.176 | 29.7% | 9.3% | 63.3% |
|  | Bagley SJ 2024 (EGFRvIII cohort) | 0 | 7 | 8 | 73 | 34.0% | 10.3% | 69.9% | 4.3 | 4.3 | 3.178 | 65.6 | 20.347 | 2.907 | -0.864 | 0.388 | 29.7% | 9.3% | 63.3% |
|  | Gu J 2025 | 3 | 6 | 8 | 74 | 26.5% | 6.6% | 64.9% | -3.2 | 3.2 | 3.809 | 68.8 | 22.467 | 3.210 | -1.224 | 0.221 | 29.7% | 9.3% | 63.3% |
|  | Gust J 2025 | 0 | 4 | 8 | 76 | 32.5% | 9.3% | 69.4% | 2.9 | 2.9 | 3.442 | 67.3 | 21.426 | 3.061 | -0.925 | 0.355 | 29.7% | 9.3% | 63.3% |
|  | Barish ME 2025 | 0 | 4 | 8 | 76 | 32.5% | 9.3% | 69.4% | 2.9 | 2.9 | 3.442 | 67.3 | 21.426 | 3.061 | -0.925 | 0.355 | 29.7% | 9.3% | 63.3% |
|  | Vitanza NA 2025 | 0 | 21 | 8 | 59 | 37.4% | 13.3% | 69.9% | 7.7 | **7.7** | 2.321 | 59.3 | 17.186 | 2.455 | -0.744 | 0.457 | 29.7% | 9.3% | 63.3% |
|  | Bagley SJ 2025 (bivalent CAR) | 18 | 18 | 8 | 62 | 22.6% | 8.3% | 48.5% | -7.1 | **7.1** | 1.414 | 50.9 | 14.244 | 2.035 | -2.059 | 0.039 | 29.7% | 9.3% | 63.3% |
| Immune effector cell-associated neurotoxicity syndrome (ICANS) | Brown CE 2022 | 0 | 6 | 6 | 53 | 13.6% | 3.3% | 42.3% | 0.7 | 0.7 | 1.989 | 63.5 | 13.708 | 2.742 | -2.356 | 0.018 | 12.9% | 3.6% | 37.0% |
|  | Lin FY 2024 | 7 | 11 | 6 | 48 | 6.5% | 2.1% | 18.3% | -6.4 | **6.4** | 0.000 | 0.0 | 0.856 | 0.171 | -4.460 | 0.000 | 12.9% | 3.6% | 37.0% |
|  | Lin Q 2021 | 0 | 3 | 6 | 56 | 12.5% | 2.9% | 40.2% | -0.4 | 0.4 | 2.075 | 65.4 | 14.439 | 2.888 | -2.461 | 0.014 | 12.9% | 3.6% | 37.0% |
|  | Bagley SJ 2024 (EGFRvIII cohort) | 0 | 7 | 6 | 52 | 13.9% | 3.4% | 42.8% | 1.0 | 1.0 | 1.955 | 62.9 | 13.476 | 2.695 | -2.334 | 0.020 | 12.9% | 3.6% | 37.0% |
|  | Gu J 2025 | 0 | 6 | 6 | 53 | 13.6% | 3.3% | 42.3% | 0.7 | 0.7 | 1.989 | 63.5 | 13.708 | 2.742 | -2.356 | 0.018 | 12.9% | 3.6% | 37.0% |
|  | Vitanza NA 2025 | 0 | 21 | 6 | 38 | 16.7% | 4.5% | 45.8% | 3.8 | 3.8 | 1.552 | 54.7 | 11.030 | 2.206 | -2.189 | 0.029 | 12.9% | 3.6% | 37.0% |
|  | Li X 2026 | 0 | 5 | 6 | 54 | 13.3% | 3.2% | 41.8% | 0.4 | 0.4 | 2.022 | 64.2 | 13.950 | 2.790 | -2.383 | 0.017 | 12.9% | 3.6% | 37.0% |
