# Supplementary material for: Efficacy of CAR‐T‐Cell‐Based Immunotherapies in Patients With Glioma: A Systematic Review and Meta‐Analysis
Source: J Cell Mol Med. 2026 Sep 25;30(18):e71358. doi: 10.1111/jcmm.71358 (PMC13614879; doi:10.1111/jcmm.71358)
Supplement: Supplementary file 3 [file JCMM-30-e71358-s009.docx]

| **Section and Topic** | **Item #** | **Checklist item** | **Location where item is reported** |
| --- | --- | --- | --- |
| **TITLE** | | |  |
| Title | 1 | Identify the report as a systematic review. | **Title page: Efficacy of CAR-T cell-based immunotherapies in patients with glioma: A systematic review and meta-analysis** |
| **ABSTRACT** | | |  |
| Abstract | 2 | See the PRISMA 2020 for Abstracts checklist. | Reported in the structured Abstract (Background, Methods, Results, Conclusions) |
| **INTRODUCTION** | | |  |
| Rationale | 3 | Describe the rationale for the review in the context of existing knowledge. | **Introduction** (Paragraphs 3 & 4) |
| Objectives | 4 | Provide an explicit statement of the objective(s) or question(s) the review addresses. | Final paragraph of Introduction \| \| \| |
| **METHODS** | | |  |
| Eligibility criteria | 5 | Specify the inclusion and exclusion criteria for the review and how studies were grouped for the syntheses. | **Methods: Study Selection (Section 2.2)**  **Methods: Subgroup and Sensitivity Analysis (Section 2.5)** - for grouping by target antigen. |
| Information sources | 6 | Specify all databases, registers, websites, organisations, reference lists and other sources searched or consulted to identify studies. Specify the date when each source was last searched or consulted. | **Methods: Search Strategy (Section 2.1)** - "A systematic search of the PubMed/MEDLINE, Embase, Scopus, Web of Science, and ProQuest databases was performed. The date limit was set from January 1, 1992, to June 3, 2026." and "...manually screened the reference lists..." |
| Search strategy | 7 | Present the full search strategies for all databases, registers and websites, including any filters and limits used. | **Methods: Search Strategy (Section 2.1)** - "The specific search strategies for each database are summarized in Supplementary Table 1." |
| Selection process | 8 | Specify the methods used to decide whether a study met the inclusion criteria of the review, including how many reviewers screened each record and each report retrieved, whether they worked independently, and if applicable, details of automation tools used in the process. | **Methods: Study Selection (Section 2.2)** - "...independently reviewed by two authors (Sh. Sh. D. and H. Sh.)... Any discrepancies during the screening process were resolved by a (S. K.)..." |
| Data collection process | 9 | Specify the methods used to collect data from reports, including how many reviewers collected data from each report, whether they worked independently, any processes for obtaining or confirming data from study investigators, and if applicable, details of automation tools used in the process. | **Methods: Data Extraction and Risk of Bias Assessment (Section 2.3)** - "Two reviewers independently extracted data from the eligible full-text reports using a standardized data-extraction form." |
| Data items | 10a | List and define all outcomes for which data were sought. Specify whether all results that were compatible with each outcome domain in each study were sought (e.g. for all measures, time points, analyses), and if not, the methods used to decide which results to collect. | **Methods: Data Extraction and Risk of Bias Assessment (Section 2.3)** - "Extracted variables included... complete response (CR), partial response (PR), stable disease (SD), progressive disease (PD), grade ≥3 adverse events, cytokine release syndrome (CRS), immune effector cell-associated neurotoxicity syndrome (ICANS), and survival outcomes where available." |
|  | 10b | List and define all other variables for which data were sought (e.g. participant and intervention characteristics, funding sources). Describe any assumptions made about any missing or unclear information. | **Methods: Data Extraction and Risk of Bias Assessment (Section 2.3)** - "Extracted variables included author, publication year, country, study design and trial phase, patient population and tumor characteristics, target antigen, CAR-T construct, route and dose of administration, Pre-treatment history, number of enrolled, treated, and outcome-evaluable participants, duration of follow-up, response criteria..." |
| Study risk of bias assessment | 11 | Specify the methods used to assess risk of bias in the included studies, including details of the tool(s) used, how many reviewers assessed each study and whether they worked independently, and if applicable, details of automation tools used in the process. | **Methods: Data Extraction and Risk of Bias Assessment (Section 2.3)** - "...assessed using the Joanna Briggs Institute (JBI) Critical Appraisal Checklist for Case Series... Two reviewers independently extracted data..." (It's implied the same process was used for risk of bias). |
| Effect measures | 12 | Specify for each outcome the effect measure(s) (e.g. risk ratio, mean difference) used in the synthesis or presentation of results. | **Methods: Meta-Analysis (Section 2.4)** - "For each study, the event proportion was calculated..." and "pooled estimates with 95% confidence intervals (CIs) were back-transformed to the proportion scale for reporting." |
| Synthesis methods | 13a | Describe the processes used to decide which studies were eligible for each synthesis (e.g. tabulating the study intervention characteristics and comparing against the planned groups for each synthesis (item #5)). | **Methods: Subgroup and Sensitivity Analysis (Section 2.5)** - "Exploratory subgroup analyses were conducted according to CAR-T target antigen or marker type..." |
|  | 13b | Describe any methods required to prepare the data for presentation or synthesis, such as handling of missing summary statistics, or data conversions. | **Methods: Meta-Analysis (Section 2.4)** - "A continuity correction of 0.5 was applied to both the event and non-event cells only when a study reported zero events or events in all participants." |
|  | 13c | Describe any methods used to tabulate or visually display results of individual studies and syntheses. | **Results: Study Characteristics (Section 3.1)** - "The characteristics of the included studies are summarized in Table 1." and **Results: Meta-Analysis (Section 3.3)** - "Forest plots were generated..." (Figures 2-8). |
|  | 13d | Describe any methods used to synthesize results and provide a rationale for the choice(s). If meta-analysis was performed, describe the model(s), method(s) to identify the presence and extent of statistical heterogeneity, and software package(s) used. | **Methods: Meta-Analysis (Section 2.4)** - "...proportions were analyzed on the logit scale. Random-effects models were fitted using restricted maximum likelihood (REML)... Between-study heterogeneity was assessed using Cochran's Q statistic, I², and τ²... using Stata/SE version 17.0..." |
|  | 13e | Describe any methods used to explore possible causes of heterogeneity among study results (e.g. subgroup analysis, meta-regression). | **Methods: Subgroup and Sensitivity Analysis (Section 2.5)** - "Exploratory subgroup analyses were conducted according to CAR-T target antigen..." |
|  | 13f | Describe any sensitivity analyses conducted to assess robustness of the synthesized results. | **Methods: Subgroup and Sensitivity Analysis (Section 2.5)** - "Leave-one-out sensitivity analyses were performed..." |
| Reporting bias assessment | 14 | Describe any methods used to assess risk of bias due to missing results in a synthesis (arising from reporting biases). | **Methods: Publication Bias Assessment (Section 2.6)** - "Potential small-study effects... were assessed using contour-enhanced funnel plots and Egger's regression test." |
| Certainty assessment | 15 | Describe any methods used to assess certainty (or confidence) in the body of evidence for an outcome. | **Not applicable. No formal method, such as GRADE, was used to assess the certainty or confidence in the body of evidence for each outcome.** |
| **RESULTS** | | |  |
| Study selection | 16a | Describe the results of the search and selection process, from the number of records identified in the search to the number of studies included in the review, ideally using a flow diagram. | **Results: Study Characteristics (Section 3.1)** - "A total of 23 studies met the eligibility criteria..." (Refer to **Figure 1** for the flow diagram). |
|  | 16b | Cite studies that might appear to meet the inclusion criteria, but which were excluded, and explain why they were excluded. | **Results: Study Characteristics (Section 3.1)** |
| Study characteristics | 17 | Cite each included study and present its characteristics. | **Results: Study Characteristics (Section 3.1)** - Summarized in text and detailed in **Table 1**. References 25-46 are cited. |
| Risk of bias in studies | 18 | Present assessments of risk of bias for each included study. | **Results: Quality Assessment (Section 3.2)** - "The mean number of affirmative responses was 8.9 out of 10... Detailed JBI item-level assessments are provided in Supplementary Table 2." |
| Results of individual studies | 19 | For all outcomes, present, for each study: (a) summary statistics for each group (where appropriate) and (b) an effect estimate and its precision (e.g. confidence/credible interval), ideally using structured tables or plots. | **Results: Meta-Analysis (Section 3.3)** - Data is presented in forest plots (Figures 2-8) and **Table 2**. |
| Results of syntheses | 20a | For each synthesis, briefly summarise the characteristics and risk of bias among contributing studies. | **Results: Meta-Analysis (Section 3.3)** - The text preceding each forest plot summarises the number of reports and participants. Risk of bias is summarised globally in Section 3.2. |
|  | 20b | Present results of all statistical syntheses conducted. If meta-analysis was done, present for each the summary estimate and its precision (e.g. confidence/credible interval) and measures of statistical heterogeneity. If comparing groups, describe the direction of the effect. | **Results: Meta-Analysis (Section 3.3)** - Pooled proportions with 95% CIs and I² values are reported for all outcomes (e.g., "The pooled OR proportion was 16.9% (95% CI, 8.0%–32.2%), with moderate heterogeneity (I² = 55.8%)"). |
|  | 20c | Present results of all investigations of possible causes of heterogeneity among study results. | **Results: Subgroup Analysis (Section 3.4)** - Presented in **Table 2**. |
|  | 20d | Present results of all sensitivity analyses conducted to assess the robustness of the synthesized results. | **Results: Sensitivity Analysis (Section 3.5)** - "Leave-one-out sensitivity analyses indicated that no individual report materially altered the overall direction or interpretation... Full numerical results are provided in Supplementary Table 3..." |
| Reporting biases | 21 | Present assessments of risk of bias due to missing results (arising from reporting biases) for each synthesis assessed. | **Results: Publication Bias (Section 3.6)** - Egger's test results are presented for OR, CR, PR, SD, and PD. |
| Certainty of evidence | 22 | Present assessments of certainty (or confidence) in the body of evidence for each outcome assessed. | **Not applicable. Certainty-of-evidence ratings were not produced because no formal certainty-assessment framework was applied.** |
| **DISCUSSION** | | |  |
| Discussion | 23a | Provide a general interpretation of the results in the context of other evidence. | **Discussion** (Paragraphs 1, 7, 8, 9, 10) |
|  | 23b | Discuss any limitations of the evidence included in the review. | **Discussion** (Paragraphs 1, 2, 3, 5) |
|  | 23c | Discuss any limitations of the review processes used. | **Discussion** (Paragraph 6, 7) |
|  | 23d | Discuss implications of the results for practice, policy, and future research. | **Conclusions and Future Perspective** (Entire section) |
| **OTHER INFORMATION** | | |  |
| Registration and protocol | 24a | Provide registration information for the review, including register name and registration number, or state that the review was not registered. | **Abstract** and **Methods: Search Strategy (Section 2.1)** - "The protocol was registered prospectively with PROSPERO (CRD42022373297)." |
|  | 24b | Indicate where the review protocol can be accessed, or state that a protocol was not prepared. | **Methods: Search Strategy (Section 2.1)**  (<https://www.crd.york.ac.uk/PROSPERO/view/CRD42022373297>) |
|  | 24c | Describe and explain any amendments to information provided at registration or in the protocol. | The study was registered in PROSPERO (CRD42022373297). The following amendments were made to the original protocol: (1) the scope was narrowed from 'cell‑based immunotherapies' to specifically 'CAR‑T cell‑based immunotherapies' to reflect the focus of the included studies. All changes have been clearly described in the Methods section and highlighted in the revised manuscript. These amendments were made to align the methodology with the available evidence and do not affect the validity of the findings. |
| Support | 25 | Describe sources of financial or non-financial support for the review, and the role of the funders or sponsors in the review. | **Funding** **section** |
| Competing interests | 26 | Declare any competing interests of review authors. | **Declaration of competing interest** and **Conflict of interest section** |
| Availability of data, code and other materials | 27 | Report which of the following are publicly available and where they can be found: template data collection forms; data extracted from included studies; data used for all analyses; analytic code; any other materials used in the review. | **Data availability section** |

*From:*  Page MJ, McKenzie JE, Bossuyt PM, Boutron I, Hoffmann TC, Mulrow CD, et al. The PRISMA 2020 statement: an updated guideline for reporting systematic reviews. BMJ 2021;372:n71. doi: 10.1136/bmj.n71. This work is licensed under CC BY 4.0. To view a copy of this license, visit <https://creativecommons.org/licenses/by/4.0/>
