# Supplementary material for: Efficacy of CAR‐T‐Cell‐Based Immunotherapies in Patients With Glioma: A Systematic Review and Meta‐Analysis
Source: J Cell Mol Med. 2026 Sep 25;30(18):e71358. doi: 10.1111/jcmm.71358 (PMC13614879; doi:10.1111/jcmm.71358)
Supplement: Supplementary file 4 [file JCMM-30-e71358-s011.docx]

| Supplementary Table S2. Joanna Briggs Institute (JBI) Critical Appraisal Checklist results for included studies. This table presents detailed item-level quality assessment results for all 23 included studies across 10 methodological domains, including clarity of inclusion criteria, reliability of condition ascertainment, consecutive inclusion, demographic reporting, outcome reporting, follow-up, study setting, and statistical analysis. | | | | | | | | | | | | |
| --- | --- | --- | --- | --- | --- | --- | --- | --- | --- | --- | --- | --- |
| **Study (year)** | **Design / phase** | **Q1 Clear inclusion criteria** | **Q2 Condition measured reliably** | **Q3 Valid condition identification** | **Q4 Consecutive inclusion** | **Q5 Complete inclusion** | **Q6 Participant demographics** | **Q7 Clinical information** | **Q8 Outcomes / follow-up** | **Q9 Site / clinic information** | **Q10 Appropriate statistics** | **Yes responses (0–10)** |
| Ahmed, N. (2017) | Phase 1 dose-escalation | Yes | Yes | Yes | Unclear | Yes | Yes | Yes | Yes | Yes | Yes | **9** |
| Brown, Ch. (2024) | Phase 1, single-arm | Yes | Yes | Yes | Unclear | Yes | Yes | Yes | Yes | Yes | Yes | **9** |
| Brown, C. (2022) | Phase 1, single-arm | Yes | Yes | Yes | Unclear | Yes | Yes | Yes | Yes | Yes | Yes | **9** |
| Goff, S. (2019) | Phase 1 dose-escalation | Yes | Yes | Yes | Unclear | Yes | Yes | Yes | Yes | Yes | Yes | **9** |
| Lim, J. (2021) | Open-label phase I/IIa | Yes | Yes | Yes | Unclear | Yes | Yes | Yes | Yes | Yes | Yes | **9** |
| Lin, F. Y. (2024) | Phase 1 | Yes | Yes | Yes | Unclear | Yes | Yes | Yes | Yes | Yes | Yes | **9** |
| Lin, Q. (2021) | First-in-human phase 1 | Yes | Yes | Yes | Unclear | Yes | Yes | Yes | Yes | Yes | Yes | **9** |
| Monje, M. (2025) | Phase 1 dose-escalation | Yes | Yes | Yes | Unclear | Yes | Yes | Yes | Yes | Yes | Yes | **9** |
| O'Rourke, D. M. (2017) | Phase 1 | Yes | Yes | Yes | Unclear | Yes | Yes | Yes | Yes | Yes | Yes | **9** |
| Vitanza, N. A. (2023) | Phase 1 interim analysis | Yes | Yes | Yes | Unclear | Unclear | Yes | Yes | Yes | Yes | Yes | **8** |
| Bagley, S. (2024) | Phase 1, single-arm | Yes | Yes | Yes | Unclear | Yes | Yes | Yes | Yes | Yes | Yes | **9** |
| Choi, B. D. (2024) | First-in-human phase 1 | Yes | Yes | Yes | Unclear | Yes | Yes | Yes | Yes | Yes | Yes | **9** |
| Brown, C. E. (2015) | First-in-human phase 1 | Yes | Yes | Yes | Unclear | Yes | Yes | Yes | Yes | Yes | Yes | **9** |
| Gu, J. (2025) | Single-arm clinical study | Yes | Yes | Yes | Unclear | Yes | Yes | Yes | Yes | Yes | Yes | **9** |
| Gust, J. (2025) | Completed phase 1 | Yes | Yes | Yes | Unclear | Yes | Yes | Yes | Yes | Yes | Yes | **9** |
| Liu, Z. (2023) | Phase 1 | Yes | Yes | Yes | Unclear | Yes | Yes | Yes | Yes | Yes | Yes | **9** |
| Barish, M. E. (2025) | First-in-human phase 1 | Yes | Yes | Yes | Unclear | Yes | Yes | Yes | Yes | Yes | Yes | **9** |
| Vitanza, N. A. (2025) | Completed phase 1 | Yes | Yes | Yes | Unclear | Yes | Yes | Yes | Yes | Yes | Yes | **9** |
| Majzner, R. G. (2022) | Phase 1 dose-escalation | Yes | Yes | Yes | Unclear | Yes | Yes | Yes | Yes | Yes | Yes | **9** |
| Bagley, S. J. (2024) | Phase 1 interim analysis | Yes | Yes | Yes | Unclear | Yes | Yes | Yes | Yes | Yes | Yes | **9** |
| Bagley, S. J. (2025) | Completed phase 1 | Yes | Yes | Yes | Unclear | Yes | Yes | Yes | Yes | Yes | Yes | **9** |
| Tang, O. Y. (2022) | Translational analysis of phase 1 cohort | Yes | Yes | Yes | Unclear | No | Yes | Yes | Yes | Yes | Unclear | **7** |
| Li, X. (2026) | First-in-human phase 1 | Yes | Yes | Yes | Unclear | Yes | Yes | Yes | Yes | Yes | Yes | **9** |
